# Kilometer-scale convection-allowing model emulation using generative diffusion modeling

**DOI:** 10.1126/sciadv.adv0423

**Published:** 2026-01-30

**Authors:** Jaideep Pathak, Yair Cohen, Piyush Garg, Peter Harrington, Noah Brenowitz, Dale Durran, Morteza Mardani, Arash Vahdat, Shaoming Xu, Karthik Kashinath, Michael Pritchard

**Affiliations:** ^1^ NVIDIA Corporation, Santa Clara, CA, USA.; ^2^ University of Minnesota, Minneapolis, MN, USA.

## Abstract

Storm-scale convection-allowing models (CAMs) explicitly resolve convective dynamics within the atmosphere to predict the evolution of thunderstorms and mesoscale convective systems that result in damaging extreme weather. Deep learning models have, thus far, not proven skillful in this regime of kilometer-scale atmospheric simulation, despite being competitive at coarser resolutions with state-of-the-art global, medium-range weather forecasting. We present a generative diffusion model called StormCast, which emulates the High-Resolution Rapid Refresh (HRRR)—National Oceanic and Atmospheric Administration’s state-of-the-art 3-kilometer operational CAM. StormCast autoregressively predicts 99 state variables at the kilometer scale using a 1-hour time step, with dense vertical resolution in the atmospheric boundary layer, conditioned on 26 synoptic variables. We show successfully learned kilometer-scale dynamics including competitive 1- to 6-hour forecast skill for composite radar reflectivity alongside physically realistic convective cluster evolution, moist updrafts, and cold pool morphology. These results present opportunities for improving kilometer-scale regional ML weather prediction and future climate hazard dynamical downscaling.

## Teaser

Generative diffusion models emulate storm-scale weather, rivaling operational forecasts at the kilometer scale.

## INTRODUCTION

Forecasting kilometer-scale weather provides crucial information about extreme weather phenomena. At these scales, convection is organized into coherent systems such as mesoscale convective systems (MCSs), supercells, squall lines, derechos, and hurricanes (*1*, *2*), causing extreme winds, flooding, hailstorms, and tornadoes that can surpass annual damages of $50 billion in the US alone (*3*).

Reliable numerical weather prediction of mesoscale phenomena, even in regional domains, incurs substantial computational costs due to the kilometer-scale spatial resolution needed to represent the underlying fluid-dynamic motions (*4*, *5*). Several nations support such large modeling efforts, which, when coupled with data assimilation, can produce skillful forecasts of mesoscale weather (*6*–*8*). The massive computational expense of these regional models—often referred to as convection-allowing models (CAMs) or convection-permitting models—prohibits their adoption in many countries while forcing a trade-off between the resolution and the ensemble size available for probabilistic prediction for those that can afford it. The latency of producing, postprocessing, and serving such kilometer-scale forecasts can prohibit the potential benefits of these modeling systems in early warning of severe storms, which are provided on the order of hours ahead of a coming event. A recent commentary (*9*) highlights the challenge (*10*) of serving numerical weather forecasts in an ever-higher resolution ensemble (*11*) in an operational setting, where forecasts must be generated faster than the weather unfolds within the constraints of computational scaling laws (*12*).

At lower resolutions, machine learning (ML) models trained on data-assimilating global models have emerged as data-driven prediction systems that can exceed the skill of numerical weather models. These ML models, with a spatial resolution of about 30 km and a typical temporal resolution of 6 hours, have obtained up to four orders of magnitude speed-ups while maintaining comparable skill (*13*–*18*). As such, they present a pathway to increasing the size of the ensemble of their counterpart, numerical global weather models that are used for operational forecasts (*19*, *20*). Because they emulate the total tendency of a data-assimilating system, or reanalysis, rather than the tendency of its physical modeling component alone, such approaches have the potential to outperform conventional prediction models.

If ML models can also be demonstrated to successfully emulate kilometer-scale atmospheric dynamics using high-resolution reanalysis, they could be used by operational centers to provide improved forecasts or better serve time-critical high-resolution ensemble forecasts economically.

We posit that this task is not trivial. The two main challenges are the difference in the dominant forces in the fluid dynamics at the kilometer scale and the temporal resolution of kilometer-scale data available on national domain sizes. The dynamics of the atmosphere at the kilometer scale is far more complicated than at the 30-km scale, where atmospheric dynamics are constrained by hydrostatic balance in all vertical columns and geostrophic balance in midlatitudes. Such large-scale circulations have a predictability window of about 2 weeks (*21*). However, at the kilometer scale, atmospheric dynamics often deviate from these balances leading to strong, buoyancy-driven, convective motions. Thus, at the kilometer scale, atmospheric motions are akin to three-dimensional (3D turbulence, which is inherently less predictable (*22*, *23*). Numerical modeling at the kilometer scale is notably harder than at 30-km scale. Specifically, at these higher resolutions, nonhydrostatic numerical dynamical cores are used to capture the vertical accelerations in moist convection; vertical motions can rival horizontal flows, and the subgrid physics packages in these models typically include many more species of precipitating hydrometeors and their interactions (*5*, *24*).

The second challenge relates to an important observation that limits the size of deterministic ML model time steps. Although not bound by constraints like the Courant-Friedrichs-Lewy (CFL) condition, most deterministic ML models will predict the ensemble mean and filter out any spatial scales not predictable at that lead time producing unrealistically smooth forecasts. Unfortunately, the most readily available and quality-controlled national high-resolution and global low-resolution weather datasets are available at a 1-hour sampling interval. At this timescale, only spatial scales larger than 10 to 20 km are predictable (*25*, *26*) so deterministic models trained with such data will not produce realistic variability at the kilometer scale. Therefore, the available data for regional convective-scale ML training are undersampled in time compared to global reanalyses such as ERA5 (*27*). Consistent with this view, ref. (*28*) explored 10-km resolution, regional deterministic ML emulation, resulting in forecasts with skill only for several smooth variables but lacking the fine spatial scales for variables related to turbulence and storm formation processes, which are more stochastic.

On the one hand, if subhourly data are available, (*29*) recently showed that it is possible to emulate kilometer-scale tendencies of a physics-based model, such as over subsets of the US when such data are archived from bespoke forecasting systems. Here, our interest lies instead in finding what is possible when targeting the total tendencies of a joint modeling/observation system, intentionally at the frequency of its native data assimilation, and in datasets that can readily scale to complete national coverage. Together, these constraints require innovative ML techniques to overcome the challenge of learning convective dynamics using data at 1-hour time frequency.

Generative ML models have shown promising results for kilometer-scale stochastic fields in both downscaling [cf. refs. (*30*, *31*)] and nowcasting [c.f. refs. (*32*, *33*)] as well as skillful ensemble forecasting at a global 30-km resolution (*18*). Some ML models have demonstrated skillful, high-resolution probabilistic outlooks summarizing statistical outcomes (*34*–*36*) of precipitation and near-surface meteorological quantities, often directly from observational input data. Although such parsimonious ML models have shown remarkable abilities, the role of an operational CAM in meteorology extends beyond generating skillful quantitative estimates of precipitation and near-surface meteorological variables. CAMs are useful to meteorologists for tracking the evolution and structure of thunderstorms, monitoring the convective mode, and separating the stochastic from the deterministic evolution of convective organization across multiple interacting phenomena. For instance, estimating the likelihood of tornado formation tends to be based on knowledge of the predicted convective mode and storm structure (*37*). We hypothesize that predicting the complex evolution of storm-scale phenomena requires generative modeling of the full atmospheric state.

These considerations motivate our work on realistic emulation of convective dynamics by directly predicting the total temporal evolution of a dense kilometer-scale state vector within a data-assimilating kilometer-scale prediction system. They also distinguish it from prior works on regional forecasting, which either do not provide a faithful representation of physical convective processes or emulate only the tendencies of a physical model using data over a limited domain available at subhourly frequency. In this work, we present StormCast, a generative ML emulator of kilometer-scale, hourly atmospheric dynamics. StormCast is trained from an operational, radar-assimilating kilometer-scale weather model that resolves a large range of convective motions.

To capture the stochastic nature of the atmosphere at these scales, we use a generative diffusion model (*38*–*41*). References (*18*, *30*) have successfully demonstrated the use of generative diffusion models for medium-range ensemble weather forecasting and kilometer-scale downscaling, respectively. Reference (*18*) models the conditional distribution of future atmospheric states given current and past states using a diffusion model. Reference (*30*) models the distribution of kilometer-scale variables such as surface wind, temperature, and precipitation conditioned on the synoptic-scale state of the atmosphere using a combination of a stochastic diffusion model and a deterministic model in a two-step process. Both works closely follow the diffusion model formulation presented in ref. (*42*). We adopt many of the same design choices as refs. (*30*, *42*) in training and sampling our diffusion model. The design choices of ref. (*42*) guide our architecture, scaling of inputs to the neural network, training noise schedule, and inference sampling. We also use a combination of a deterministic model and a stochastic diffusion model following ref. (*30*).

Our key results show that skillful emulation of CAMs is possible using generative diffusion. Its ease of inference allows us to generate ensembles and produce probability matched mean (PMM) forecasts from StormCast ensembles. Both fractions skill score (FSS) (*43*, *44*) and root mean square error (RMSE) of StormCast-PMM for composite radar reflectivity show comparable or even higher skill than High-Resolution Rapid Refresh (HRRR) forecasts at lead times of up to 6 hours, at thresholds of 20, 30, and 40 dBZ indicating light, light-to-moderate, and moderate rainfall. StormCast outputs also exhibit physically consistent convective dynamics. Moreover, StormCast produces a vertical structure at the kilometer-scale that is consistent with the underlying physics for convective motions and implies that a latent representation of cloud processes is required for generating plausible moist updrafts and cold pools.

## RESULTS

We begin with a series of case studies of forecasting individual convective events, then discuss StormCast’s statistical forecast skill, and lastly analyze the multivariate relationships underlying its generated convection.

### Case studies

**Fig. 1.**
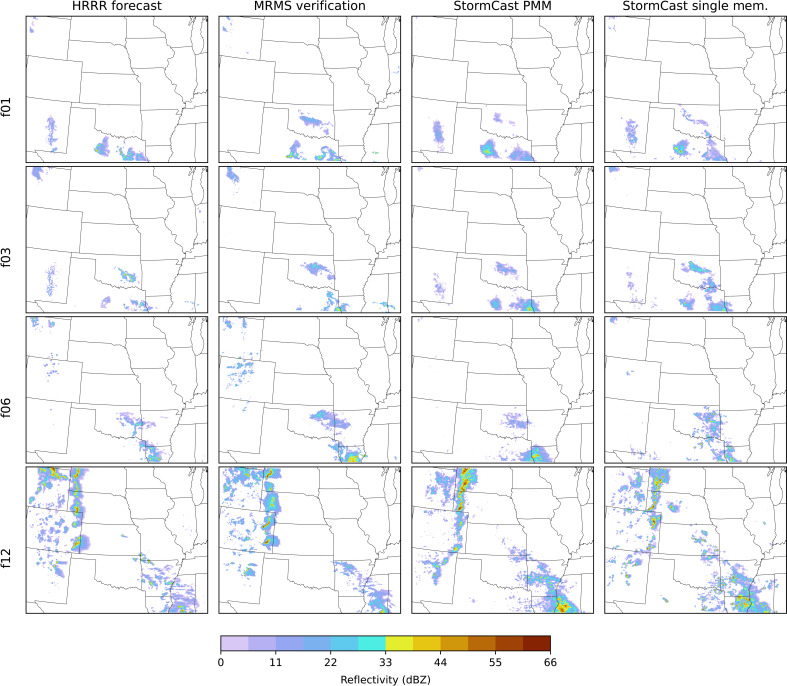
Example forecast of composite radar reflectivity generated by StormCast and HRRR compared with observed verification data from the MRMS network. From left to right, the columns show the HRRR forecast, the corresponding MRMS observation, the PMM of a five-member StormCast ensemble forecast, and one of the StormCast ensemble members. The HRRR and StormCast forecasts were initialized at 29 May 2024 12:00 UTC. The StormCast forecasts were initialized using the HRRR analysis at the initialization time. The rows from top to bottom show the forecast at progressively longer lead times (1, 3, 6, and 12 hours) along with the corresponding MRMS observation at the appropriate time.

Figure 1 compares synthetic radar reflectivity from StormCast forecasts, both for a single-member and the five-member ensemble PMM, with the HRRR forecast and observations from the Multi-Radar Multi-Sensor (MRMS) (*45*) network. The forecast begins at 12:00 UTC, early morning local time, on 29 May 2024, and the general character of the HRRR and StormCast forecasts is similar to the MRMS verification throughout the full model domain. At forecast hour 1, a large reflectivity patch develops in central-east Oklahoma, which is missed by the HRRR and is represented by StormCast and StormCast-PMM. By forecast hours 6 and 9, the StormCast PMM is doing a better job of capturing the high reflectivities in the southernmost storm near the Louisiana-Mississippi border, although later, at forecast hour 12, StormCast may be overestimating its intensity. Also at hour 12, a line of high-reflectivity storms moving east from the Rocky Mountains is reasonably well forecast by both StormCast and the HRRR.

**Fig. 2.**
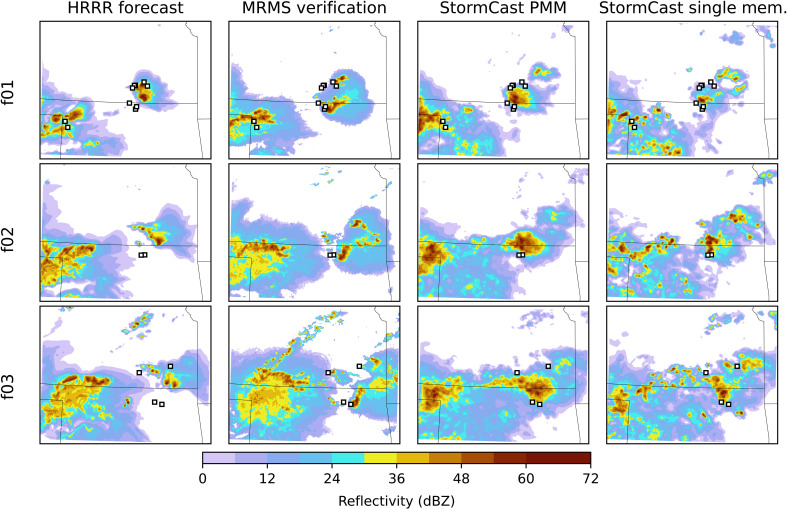
Close-up of an example forecast of composite radar reflectivity generated by StormCast and HRRR compared with observed verification data from MRMS. The HRRR and StormCast forecasts were initialized at 16 May 2024 00:00 UTC. The visualized region primarily includes the states of Kansas and Oklahoma. Storm reports from the NOAA Storm Prediction Center (SPC) within the hour preceding the forecast snapshot indicating hail are marked with square (□) shaped markers.

A close-up comparison of the distribution of convective cores in StormCast and HRRR forecasts with MRMS observations is provided in a 500-km square subdomain in Fig. 2 for a case with reports of severe hail or tornadoes. In Fig. 2, forecasts initialized at 00:00 UTC on 16 May 2024 are shown at lead times of 1, 2, and 3 hours, which covers the period in which severe hail along the Kansas-Oklahoma border was generated by an MCS with intense convective updrafts embedded in a larger region of stratiform precipitation having weaker radar returns. Both StormCast and the HRRR predict strong convective storms in the region where hail was reported, and both also capture the general character of the larger convective system farther to the west. The distribution of the individual cells in the StormCast single-member forecast is more random than that in the HRRR and the verification, although the intense radar returns are more consolidated in the StormCast PMM product. To quantify this comparison, we report the scores of HRRR, StormCast, and StormCast PMM forecasts using metrics that account for the stochastically of reflectivity maps in the following section.

### Precipitation forecasts

**Fig. 3.**
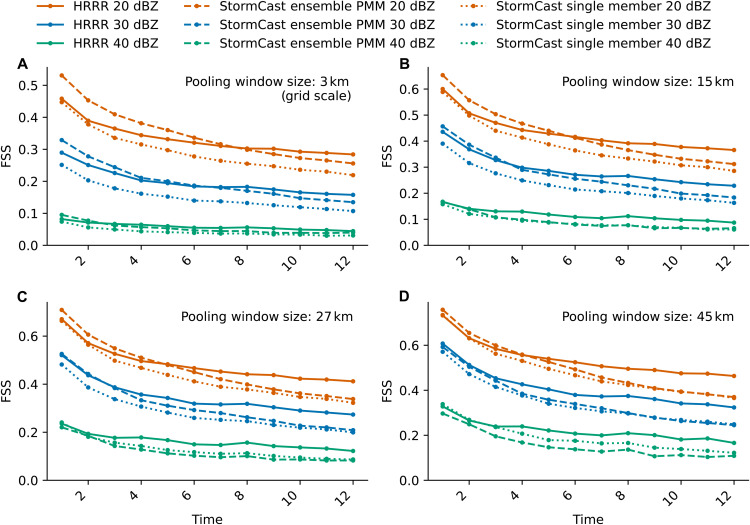
Quantitative evaluation of StormCast ensemble and single-member composite radar reflectivity forecasts relative to the HRRR. (**A** to **D**) FSS for composite radar reflectivity forecasts from StormCast’s ensemble PMM, StormCast single-member forecasts, and corresponding HRRR forecasts, evaluated at spatial scales of 3 km (A), 15 km (B), 27 km (C), and 45 km (D).

For forecasting precipitation, Fig. 3 shows that both the ensemble PMM and individual forecasts from StormCast have a competitive FSS compared to the HRRR in the first 12 hours. The FSS of composite reflectivity forecasts are evaluated at thresholds of 20 dBZ (light rain), 30 dBZ (light-to-moderate rain), and 40 dBZ (moderate rain). We compare the single-member forecasts from StormCast (dotted lines) as well as the PMM of a five-member ensemble from the StormCast model (dashed lines) against the HRRR single-member forecast baseline (solid lines).

The StormCast PMM exhibits a pronounced superiority in the most predictable FSS category, 20 dBZ (light rainfall), for small 3-km window sizes at 1- and 2-hour forecast lead times (Fig. 3A). As the pooling window size expands, this superiority diminishes, although the raw FSS scores themselves improve as expected. The PMM of StormCast’s ensemble 20-dBZ FSS forecasts remains more skillful than the HRRR out to lead times of 3 to 6 hours for pooling window sizes increasing from 3 to 45 km (Fig. 3; orange dashed versus solid). StormCast’s individual forecasts are also competitive with the HRRR at the 2-hour lead time for all pooling window sizes (Fig. 3; orange dotted versus solid).

At the 30-dBZ threshold, useful predictability (FSS larger than 0.4) exists only at 15- to 45-km pooling sizes on 1- to 3-hour lead times. Here, StormCast’s PMM ensemble skill appears to be competitive but not more skillful than the HRRR (Fig. 3, C and D; blue dashed versus solid). In contrast, individual StormCast predictions are systematically less skillful (Fig. 3; blue dotted versus solid) than the HRRR. At the 40-dBZ threshold, there is little useful predictability in either model, i.e., FSS < 0.4 at all lead times and window sizes (Fig. 3, A to D; green lines).

**Fig. 4.**
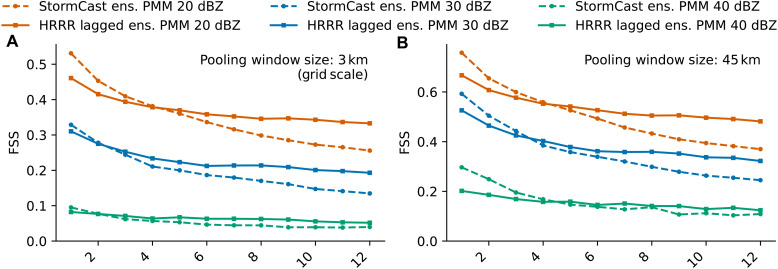
Quantitative comparison of StormCast and HRRR ensemble forecasts of composite radar reflectivity. (**A** and **B**) FSS for forecasts derived from the StormCast ensemble PMM compared to time-lagged HRRR ensemble forecasts. Evaluation at spatial scales of (A) 3 km and (B) 45 km.

It is, perhaps, expected that the PMM from the StormCast ensemble can outperform a single HRRR deterministic forecast. An ensemble of HRRR forecasts is not available for comparison, but we can create a simple ensemble using lagged forecasts (*46*). From each HRRR forecast in the evaluation set, we create a five-member lagged ensemble using forecasts initialized 1 to 4 hours before the control forecast initialization time. Each ensemble member is evaluated at a lead time aligning with the forecast verification time. Figure 4 compares the FSS of the PMM of a StormCast ensemble and that of the corresponding lagged HRRR ensemble. For brevity, we include the comparisons at just two pooling window sizes: 3 and 45 km. The HRRR lagged ensemble does improve on the single deterministic HRRR forecast at long lead times, but in the first few hours, it performs worse than the deterministic member, likely because of the substantial increase in the lag of those ensemble members initialized at the earliest times. As a consequence, the StormCast PMM for the 20-dBZ threshold outperforms the HRRR lagged ensemble over the first 3 hours at both window sizes. This advantage disappears at longer lead times, with the HRRR lagged ensemble superior to the StormCast PMM after 4 hours.

**Fig. 5.**
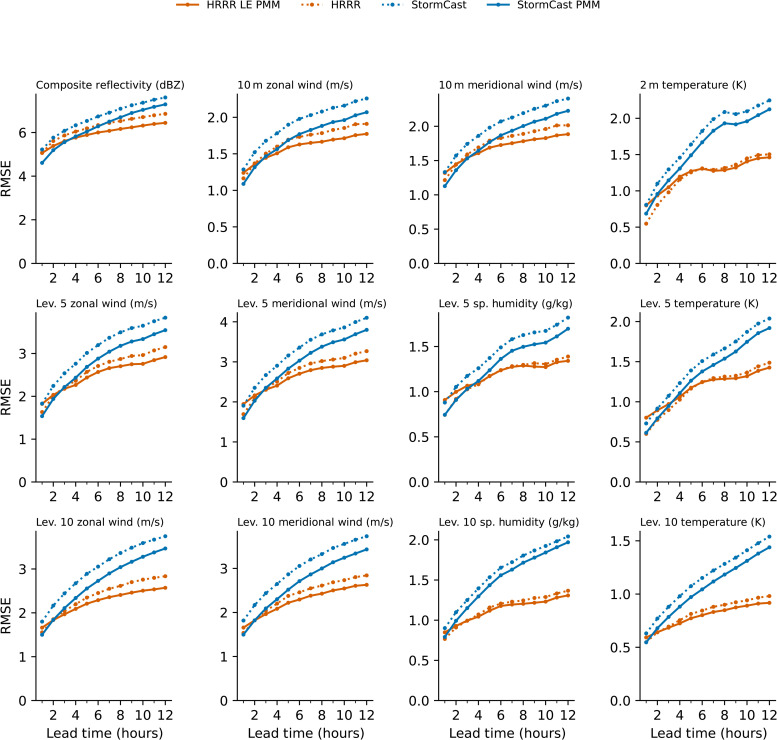
Forecast skill of a few selected variables predicted by the StormCast model compared with the HRRR model. Forecast skill is measured using the RMSE between forecasts and the verification data for composite reflectivity, 10-m wind velocity components, and 2-m temperature as well as the winds, temperature, and specific humidity at HRRR native levels 5 and 10. The verification data for all variables except the composite reflectivity are the HRRR analysis at the verification time. For composite reflectivity, the verification data are the corresponding observed reflectivity from the MRMS sensor network at the verification time. The forecast RMSE scores are averaged over 130 forecasts from 10 May to 15 June 2024 with forecasts generated four times daily at 00:00, 06:00, 12:00, and 18:00 UTC. We show the skill of the single-member HRRR forecast (orange dotted lines), a single-member StormCast forecast (blue dotted lines), the PMM of a five-member ensemble from StormCast (blue solid lines), and the PMM of an HRRR lagged ensemble forecast (orange solid lines).

Our evaluation is generalized across additional field variables in Fig. 5 by examining the RMSE relative to the HRRR analysis. In general, the StormCast individual forecasts produce systematically higher errors and error growth rates than the HRRR. However, on lead times less than 4 to 6 hours, the StormCast ensemble PMM is competitive with and capable of outperforming the HRRR. A five-member HRRR lagged ensemble PMM is more skillful than the HRRR control forecast and is included for reference to facilitate a fair comparison with the StormCast PMM. Of the variables analyzed, the StormCast PMM has less error than the HRRR baseline for radar reflectivity (1- to 5-hour lead; consistent with the FSS), and horizontal wind components near the surface (1- to 4-hour lead) as well as winds at two separate altitudes within the boundary layer (BL) (1- to 2-hour lead). Some issues with other predicted variables are also uncovered: StormCast PMM predictions are systematically less skillful than HRRR for near-surface temperature and for specific humidity and temperature on the 10th model level, which is near 2.5-km altitude. At lower altitudes, on the fifth model level near 700-m altitude, errors in specific humidity and temperature are comparable between the StormCast PMM and the HRRR baseline for short lead times.

Overall, our assessment is of encouragingly competitive precipitation forecast skill despite a relatively unsophisticated initialization used.

### Power spectra and probability distributions

**Fig. 6.**
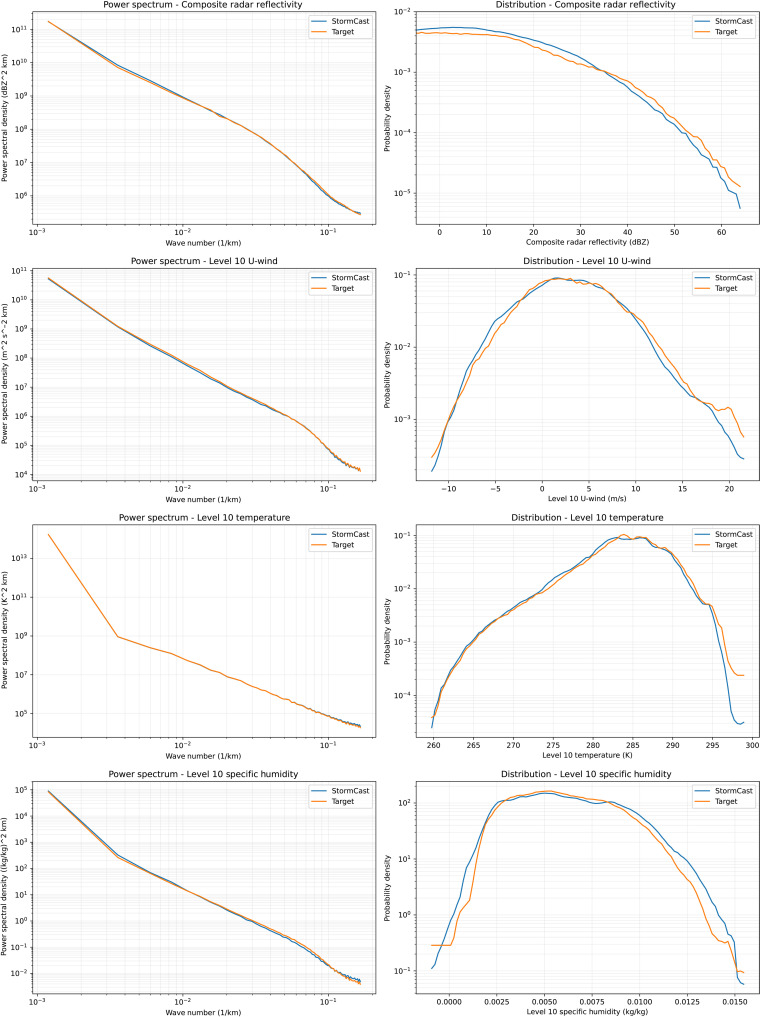
Validation of power spectra and distributions of select variables from StormCast. Azimuthally averaged power spectra (left) and distributions (right) of selected variables, comparing StormCast (blue) and the target (orange) at a lead time of 3 hours.

We next investigate the fidelity of the power spectra and probability distributions of StormCast outputs. For this task, we perform 35 separate 12-hour simulations initialized on a set of arbitrary dates during 2024. We examine one single-level variable and three variables at model level 10, which is around 1.5-km height above the ground. More variables are analyzed in the Supplementary Materials. The synoptic conditioning for StormCast is provided by a Global Forecast System (GFS) forecast. The results show that StormCast produces overall realistic spectra and probability distributions at a lead time of 3 hours (Fig. 6, left).

### Multivariate morphology of precipitating updrafts

To further assess the extent to which the model accurately represents the physics of atmospheric convection, we perform a detailed multivariate evaluation of a few individual forecasts. This serves as a meteorological counterpart to assessing the trustworthiness and explainability of ML models. We examine the model’s ability to produce vertically coherent and physically consistent convective motion, focusing on events where convective-scale dynamics should be evident. In analyzing the qualitative mesoscale dynamics, we try to ensure that the comparisons between StormCast outputs and HRRR ground truth are minimally influenced by significant deviations in synoptic-scale structure of the atmosphere. To achieve this, we use ERA5 reanalysis data as the synoptic-scale conditioning for the StormCast model in this section. In other sections of this paper that evaluate forecast skill, we use GFS forecast data as the synoptic-scale conditioning for StormCast because using reanalysis data in a forecasting context would lead to inappropriate utilization of future information.

A challenge in examining convective-scale motions in the StormCast model arises from its 1-hour time resolution, which is too coarse to observe the temporal evolution of convection (e.g., through lagged correlation analysis or videos of individual updraft evolution). Consequently, our focus is on identifying the coexistence of characteristic convective features across different channels and vertical levels within the same time frame. This approach allows us to evaluate the model’s ability to capture the spatial structure and intervariable relationships typical of convective processes, despite the temporal resolution limitations.

Moist updrafts form when air in the BL becomes warm and humid enough for rising pockets of air, called thermals, to continue ascending on their own (*47*–*49*). As these thermals rise, water vapor within them condenses, releasing latent heat. This heat increases the air’s buoyancy, propelling it further upward (*50*–*52*). The strength of these updrafts is directly related to the degree of buoyancy, which is influenced by temperature differences between the rising air and its surroundings (*50*). When these energetic thermals are strong enough to rise above the atmospheric BL, they set the stage for precipitation. This occurs as cloud droplets form and grow within the rising air, a process that can be observed through radar reflectivity measurements (*53*–*55*).

**Fig. 7.**
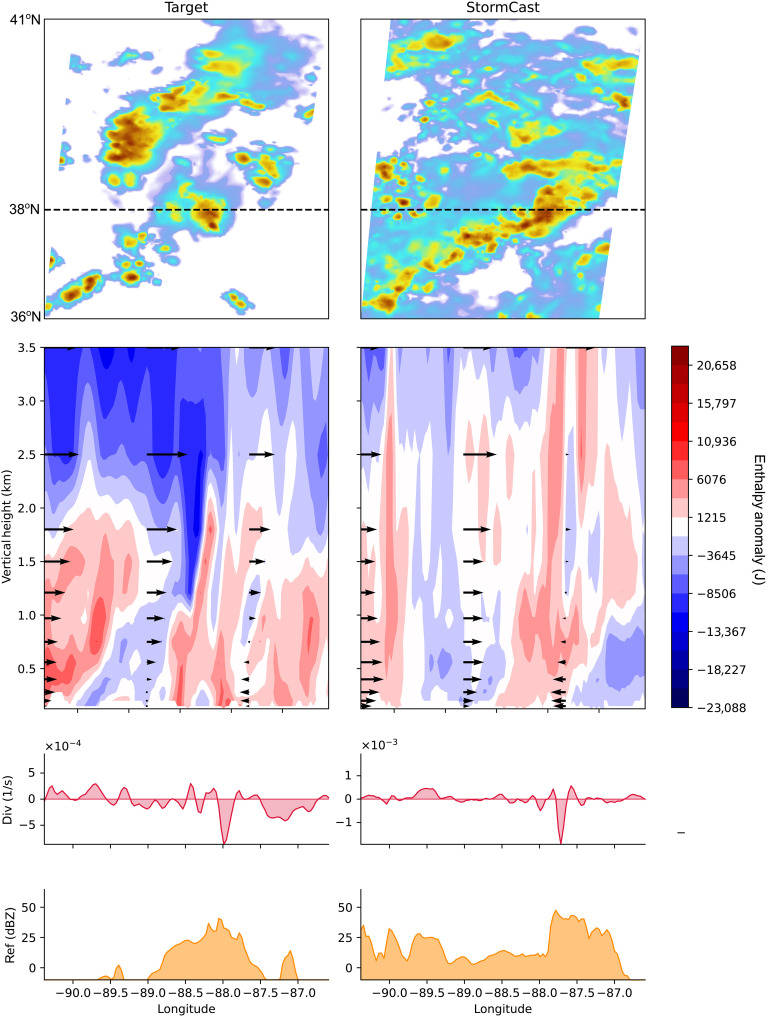
Qualitative examination of an illustrative StormCast forecast. Examination of simulated summertime convection over Illinois on 9 July 2022 at 00:00 UTC, at a 6-hour forecast lead time. (Left) HRRR model target. (Right) StormCast output. Top row: Composite radar reflectivity (planar view). The black dashed line at 38°N indicates the location of the zonal cross section displayed in the bottom rows. Second row: Zonal-vertical cross section displaying virtual moist enthalpy anomalies (contours) relative to a mean profile, overlaid with zonal wind vectors. Third row: Boundary-layer horizontal divergence vertically averaged over the first seven model layers (1-km depth) as a function of longitude. Bottom row: Zonal-vertical cross section of radar reflectivity. Color bars for reflectivity are omitted for clarity; their value ranges can be inferred from the lowest row.

Figure 7 examines such plumes from several vantage points, at a 6-hour lead time during a time period when environmental conditions were prone to convection with weak synoptic forcing on 9 July 2022 00:00 UTC, focusing on a small 4° by 4° region over the state of Illinois. The top row depicts a planar view of radar reflectivity, showing the cluster of disorganized convection that occurred in the target data, alongside a StormCast ensemble member that predicted a linear form of organized convection oriented from northwest to southeast in a similar location. The purpose is not to assess any match between kilometer-scale details of the observations and predictions—none should be expected for these spatial scales at a 6-hour lead time—but rather to anchor an analysis of StormCast’s own internally generated convective motions. A dashed line indicates a convenient latitude where convection occurred in both observations and StormCast, which defines the vertical-zonal section examined in all panels below.

The second row of Fig. 7 reveals a vertical structure along this dashed line, with shaded contours representing the anomaly of virtual moist enthalpy in the height-longitude plane, with respect to a reference profile. Moist enthalpy is a measure of the combined heat and vapor that provides buoyancy to deep convective updrafts. It is a useful quasiconserved quantity under adiabatic vertical motions and liquid-vapor phase changes of water at saturation.

To help identify the relative location of updrafts, black vectors represent zonal wind, and the third row of Fig. 7 displays the net BL divergence. This is a convenient proxy for vertical motion, which was not directly modeled in StormCast. By mass conservation, net BL divergence (convergence) corresponds to downward (upward) motion at the top of the BL because air cannot flow through Earth’s surface. Net BL divergence is estimated as the average across the model’s seven lowermost vertical levels. As with enthalpy, the horizontal mean is removed to isolate smaller-scale features from the large scale subsidence and synoptic environment.

Last, to locate the regions of precipitation, the fourth row shows a zonal transect of radar reflectivity.

Our physical expectations from observations and cloud-resolving simulations are that precipitation should spatially colocate with intense updrafts and positive enthalpy anomalies protruding above the BL. This is true of the target data (left column), where peak radar reflectivity at −88°E coincides with a prominent implicit updraft (i.e., negative net BL divergence anomaly; low-level convergence) at −87.7°E and a positive enthalpy anomaly that extends from the BL up to 3-km altitude, at the same longitude.

Our expectations are also confirmed in StormCast outputs (second column from left). StormCast predicts peak radar anomalies to occur half a degree farther east, near −88°E. A negative net BL divergence anomaly and a notable positive enthalpy anomaly extending beyond 2 km in altitude occur at the same longitude. Two other meteorological conditions are also examined in the Supplementary Materials.

### Comparison with numerical convection-allowing ensemble forecasts

**Fig. 8.**
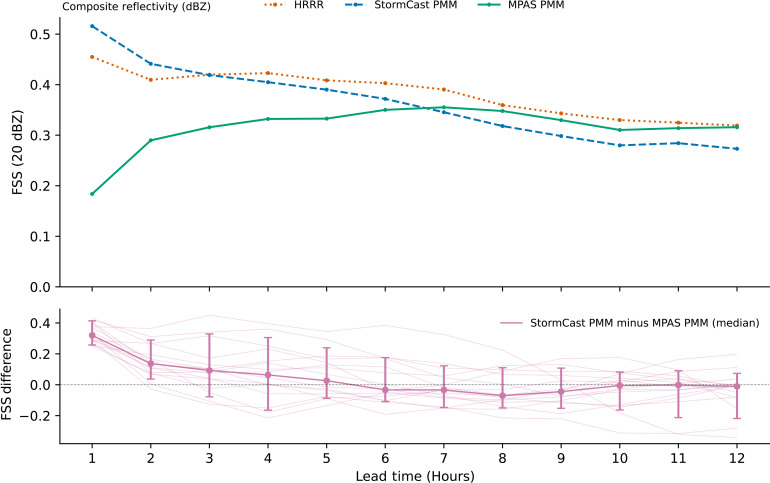
StormCast composite radar reflectivity ensemble forecast skill relative to the MPAS model. The FSS at grid scale for a threshold of 20 dBZ of StormCast five-member ensemble forecast PMM, MPAS five-member ensemble forecast PMM, and HRRR single-member forecast. The FSS was averaged over a small set of 15 forecasts. Note that the MPAS forecasts were initialized from a GEFS interpolated state and did not use a radar-assimilated initial condition (HRRR analysis) to initialize the kilometer-scale detail, unlike StormCast. The HRRR and StormCast forecasts have better skill in the first 6 hours than MPAS likely due to the difference in initialization techniques. In addition, the differences in FSS between forecast PMMs of StormCast and MPAS for each individual forecast are shown on a separate axis (thin magenta lines). The median difference (magenta bold line) as well as 10th and 90th percentiles (error bars) are also shown.

A small set of forecasts was obtained from the contribution of National Center for Atmospheric Research (NCAR) to the National Oceanic and Atmospheric Administration (NOAA) Hazardous Weather Testbed (HWT) Spring Forecasting Experiment (SFE) (*56*, *57*). The NOAA HWT SFE is an annual research campaign that explores the application of prototype CAM forecast technology to aid in predicting severe convective weather events. The NCAR submitted simulations from a configuration of its Model Prediction Across Scales (MPAS) simulator. We found 15 MPAS forecasts where we had a corresponding StormCast forecast for the same data and time. Figure 8 shows a comparison of the FSS of the StormCast five-member ensemble forecast PMM, the MPAS five-member ensemble forecast PMM, as well as an HRRR single-member forecast. The MPAS forecasts were initialized from synoptic-scale Global Ensemble Forecast System (GEFS) analysis and did not assimilate radar data at initialization. This is likely why HRRR and StormCast forecasts have better skill in the first 6 hours. We hope to repeat this analysis with a radar-assimilating ensemble CAM forecast in future work.

## DISCUSSION

The twin challenges of emulating realistic kilometer-scale 3D atmospheric dynamics and preserving fine-scale stochastic details under the constraint of relatively coarse temporal resolution (hourly) data required two innovations: (i) developing a generative ML emulator and (ii) predicting a dense atmospheric state with dozens of vertical levels. StormCast, a generative ML emulator of a kilometer-scale CAM shows that generative ML models have the potential to greatly improve and augment mesoscale meteorology in the same way that deterministic ML has improved meteorology at coarser resolutions.

We are encouraged by the following key findings:

1) StormCast’s forecasts are skillful and competitive with the operational HRRR model: Single-member forecasts of 20- and 40-dBZ reflectivity are competitive with HRRR forecasts during the first 2 hours.

2) Unlike statistical nowcasting methods that typically forecast precipitation alone, StormCast achieves this while generating multiple realizations of the evolution of mesoscale systems, cold pools, and moist updrafts with realistically colocated low-level convergence, mid-tropospheric enthalpy and radar reflectivity anomalies.

3) StormCast’s predicted mesoscale dynamics validate well on power spectra and probability distributions.

4) StormCast’s small, five-member ensemble can create an ensemble PMM with better skill than a single physics-based forecast, including by directly leveraging HRRR data assimilation through a consistent initialization. Its ensemble PMM is competitive with a time-lagged HRRR forecast during the first 4 hours.

5) Although trained with synoptic conditioning from ERA5, StormCast can be forced with deterministic GFS forecast conditioning during test time.

Together, these results imply strong potential for generative autoregressive ML as a valid alternative to traditional kilometer-scale numerical prediction, enabling applications in weather forecasting and climate downscaling (*58*–*61*) alike.

We readily admit several limitations of our model worthy of future work. StormCast was trained on a relatively small amount of data from 3.5 years of HRRR analysis. Increasing the volume of training data could improve the forecast accuracy of future models. Similarly, increasing the domain size of training could expose the model to a more diverse set of severe weather phenomena to learn from. Reanalysis datasets such as CONUS404 (*62*) offer much longer historical records and could be leveraged for training to improve model performance. We trained on forecast state data lagged 1 hour from the HRRR data assimilation cycle to allow for additional model spin-up; training directly on assimilated state data could produce complementary skill. Our evaluation period was limited to a short time interval spanning only a portion of spring of 2024; expanding skill assessment across more regions and phases of the seasonal cycle could uncover additional findings. Last, ensuring consistency in the training and testing data—both for the operational analysis version and for conditioning—could improve results.

Preliminary evaluations indicate that StormCast ensembles are underdispersive (Supplementary Materials). Better training and sampling strategies for diffusion models and incorporating sources of initial condition uncertainty could alleviate this issue. Ensemble calibration in medium-range artificial intelligence (AI) weather models has been explored in refs. (*19*, *20*, *63*). Furthermore, information about the synoptic-scale forecast uncertainty could be incorporated into StormCast by driving the StormCast model with a global ensemble forecast such as the NCEP GEFS rather than driving StormCast with a single GFS forecast. Further improving ensemble calibration could unlock the path to very large operational ML ensemble forecasts at the kilometer-scale that would otherwise incur prohibitive computational costs with current numerical models.

Further work is needed to evaluate the physical consistency and statistical skill of mesoscale ML prediction models such as StormCast. This requires a community effort in designing appropriate forecast baselines and metrics. The performance of ML models in accurately forecasting severe weather must be rigorously tested before such models can be used in an operational setting. The role of meteorologists and atmospheric scientists will be prominent in designing benchmarks for mesoscale weather forecasts, particularly in assessing forecast skill on severe weather. Leading Numerical Weather Forecast centers such as the European Center for Medium Range Weather Forecasting (ECMWF) and the NOAA have played a role as both creators of state-of-the-art ML models (*17*) and the trustworthy arbiters of the forecast quality of novel experimental ML forecast paradigms emerging from academia, industry, and operational centers. To achieve its full potential, new simulation datasets at various length scales and timescales will need to be created tailored to ML model training. We hope that the results presented in our work inspire the development of regional analysis, reanalysis, and reforecast datasets over different parts of the globe for training ML models such as StormCast.

It is important to contextualize our work relative to that of ref. (*29*) who recently showed that it is possible to learn autoregressive convective dynamics for small subregions of the US by training deterministic architectures on the tendencies of a numerical modeling system for which data happen to exist at conveniently subhourly time resolution from an archive of an operational modeling system that is sporadically spawned at times and in regions where extreme convection is predicted to be likely. Our work deviates from this in two important ways. First, we intentionally limit our attention to what is possible with a dataset that has complete US spatiotemporal coverage, with the aim of readily scaling to CONUS-wide predictions. Second, our goal is not to learn the tendencies of a numerical model but rather the tendencies of a joint modeling/observation system as close as possible to the timescale of its native data assimilation cycle. Only this affords the potential of ML to extract information from observations beyond the capacity of existing numerical simulation methods, as has proved possible using ERA5 data for global, medium-range weather predictions. Together, these constraints require us to address the challenge of learning convective dynamics using data at 1-hour time frequency.

Our current work focuses on model emulation alone. Generating a reliable weather forecast requires the additional critical step of assimilating observations to generate an estimate of the present state of the earth system, from which a forecast can be generated [c.f. ref. (*64*)]. We rely on the numerical HRRR model for state estimation; our model is therefore not an end-to-end ML-based forecast system. However, recent studies have proposed applications of generative diffusion models for data assimilation at a range of spatial scales, including at kilometer-scale resolutions (*65*–*67*). Leveraging these advances could allow for an ML-based end-to-end forecast system, which could greatly accelerate and augment operational time-critical ensemble generation at kilometer scales and beyond.

## MATERIALS AND METHODS

### Generative time-stepped diffusion modeling

Consider the coarse-resolution synoptic-scale state ${\left\{S_{t}\right\}}_{t\geq t_{0}}$ at 30 km and the initial high-resolution mesoscale condition $M_{t=t_{0}}$ at 3 km. Our goal is to forecast the high-resolution mesoscale states $M_{t}$ for $t>t_{0}$. At each time t, we have access to the coarse-resolution forecast $S_{t}$, typically available from another physics-based [e.g., Integrated Forecasting System (IFS) and GFS] or AI-based model (e.g., FourCastNet). Thus, we are motivated to learn the time-stepping distribution for time-ahead as the conditional distribution $p_{\Theta }\left(M_{t+1}|S_{t},M_{t}\right)$. Once one has access to $p_{\Theta }\left(M_{t+1}|S_{t},M_{t}\right)$, one can autoregressively sample the k-hour-ahead future for arbitrary k based on the initial condition $M_{t_{0}}$ and the coarse-resolution forecast $S_{t}$.

To learn the conditional distribution $p_{\Theta }\left(M_{t+1}|S_{t},M_{t}\right)$, we adopt denoising diffusion models due to their effective mode coverage and training stability. Specifically, we follow the approach proposed in (*30*) for learning the conditional distribution of weather data with both deterministic and stochastic dynamics. Following CorrDiff (*30*), we decompose the learning of $p_{\Theta }$ into two phases, namely, deterministic regression and stochastic diffusion. The outline of the two-step approach followed by StormCast is as follows. A deterministic model $F_{\theta }\left(M_{t},S_{t}\right)$ estimates the conditional mean $\mu_{t+1}$ of the forecast distribution. A diffusion model is trained to generate samples of a “residual” term $r_{t+1}$ from a conditional distribution $p_{\phi }\left(r_{t+1}|\mu_{t+1},M_{t}\right)$. Samples of residual $r_{t+1}$ conditioned on $\mu_{t+1}$ and $M_{t}$ generated by the diffusion model are added to the conditional mean $\mu_{t+1}$ to give a sample from the forecast distribution $M_{t+1}$. Thus, the deterministic model and the diffusion model together approximate samples from the conditional data distribution $p_{\Theta }\left(M_{t+1}|S_{t},M_{t}\right)$ parametrized by Θ={θ,ϕ}.

#### Phase 1: Deterministic regression

First, we learn a deterministic regression $F_{\theta }\left(M_{t},S_{t}\right)$ to estimate the conditional mean, namely

$$F_{\theta }\left(M_{t},S_{t}\right)=E\left[M_{t+1}|M_{t},S_{t}\right]$$ (1)

To do so, we can train a U-Net based on the paired data samples ${\left\{\left(M_{t_{i}+1},M_{t_{i}},S_{t_{i}}\right)\right\}}_{i=0}^{N}$, using a mean squared error loss as follows

$$\theta^{*}=\underset{\theta }{\text{argmin}}\;\frac{1}{N}\sum_{i=1}^{N}{\left\|M_{t_{i}+1}-F_{\theta }\left(M_{t_{i}},S_{t_{i}}\right)\right\|}_{2}^{2}$$ (2)

With the learned regression model at hand, we can form the residuals

$$r_{t+1}M_{t+1}-\mu_{t+1}\;\text{where}\;\mu_{t+1}≔F_{\theta^{*}}\left(M_{t},S_{t}\right)$$ (3)

#### Phase 2: Stochastic diffusion

**Fig. 9.**
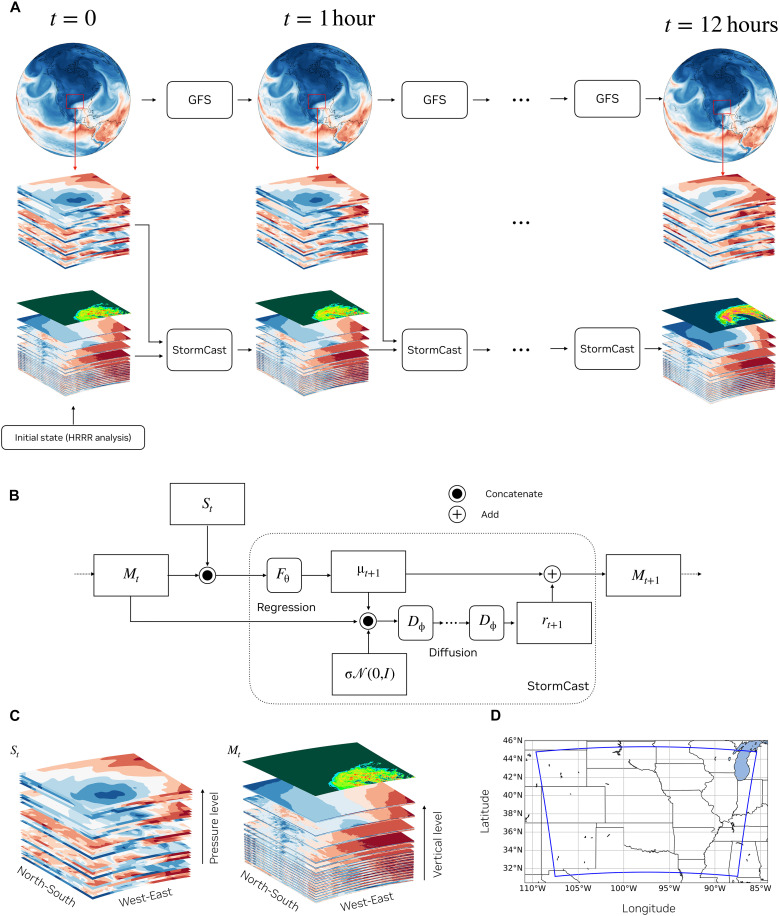
StormCast autoregressive forecast setup. (**A**) StormCast generates an autoregressive forecast starting from an initial condition generated by HRRR analysis and using an hourly synoptic scale forecast produced by the GFS model. (**B**) Illustration of how StormCast generates a kilometer-scale forecast in a two-step process. The synoptic-scale fields $S_{t}$ and the mesoscale fields $M_{t}$ at time *t* are used to generate a 1-hour ahead deterministic mean forecast $\mu_{t+1}$ using a neural network $F_{\theta }$ with a U-Net architecture. The mean forecast $\mu_{t+1}$ and the $M_{t}$ are concatenated with a latent random Gaussian noise vector and passed through a denoising diffusion model $D_{\phi }$ for a series of diffusion steps to sample an estimate of the residual forecast $r_{t+1}$, which is added to the mean $\mu_{t+1}$ to generate $M_{t+1}$ from the forecast distribution at time t+1. This process is repeated autoregressively to generate a 12-hour forecast. The synoptic-scale conditioning $S_{t}$ at each time step *t* is provided to the model via a 25-km global model—the NCEP GFS model in forecasting mode and ERA5 reanalysis in our hindcast tests. (**C**) Stacked channels representing the synoptic-scale state $S_{t}$ on a pressure-level vertical grid and interpolated to the kilometer-scale domain as well as the mesoscale state $M_{t}$ on the native HRRR model hybrid vertical grid. Refer to Table 1 for the full channel set. The spatial extent of the domain of operation of the StormCast model is illustrated in (**D**) with a blue bounding box. The domain size is 1536 km by 1920 km.

After forming the residuals, we train a diffusion model to learn the conditional distribution $p\left(r_{t+1}|\mu_{t+1},M_{t}\right)$. After training the diffusion model, samples from the desired conditional distribution $p\left(M_{t+1}|M_{t},S_{t}\right)$ are obtained by combining the deterministic prediction and stochastic diffusion sample as $r_{t+1}+\mu_{t+1}$. Figure 9B illustrates the steps involved in generating a 1-hour forecast using StormCast. Figure 9A shows how an autoregressive forecast is generated using a synoptic scale model and StormCast.

### Diffusion model configuration

Diffusion models are designed to model a given data distribution $p_{\text{data}}\;\left(x\right)$ by training a denoising network $D_{\phi }\left(z,\sigma \right)$ parametrized by ϕ, which accepts as inputs a noisy vector **z** and the noise level σ. The denoising network is trained to remove the Gaussian noise added to the training data samples during the training process. The exact form of the training objective typically varies between different formulations of the diffusion model. A diffusion process is defined via a stochastic differential equation (SDE) or, in alternate formulations, an ordinary differential equation (ODE). In the forward process, samples from the data distribution are continuously noised by adding varying amounts of Gaussian distributed random noise until the data sample is completely transformed into random noise. In the reverse process, a sample of Gaussian random noise is used as a starting point for a diffusion SDE or ODE. It has been shown in several theoretical formulations [c.f. (*38*, *41*, *42*, *68*–*70*)] that the trained denoising network can be used as part of a diffusion SDE or ODE to recover samples from the training data distribution in the reverse process starting from Gaussian random noise. Diffusion models can also be used in a conditional setting where the model is trained to generate the conditional distribution $p_{\text{data}}\;\left(x|y\right)$ using a denoising network as part of a stochastic diffusion process.

In designing our diffusion model, we closely follow the architecture, design, and training protocol proposed in ref. (*42*), which carefully reformulates the training objective, noise distributions, and sampling procedures to improve training stability and sample quality. Our goal, as previously stated, is to generate the conditional distribution $p\left(r_{t+1}|\mu_{t+1},M_{t}\right)$. Following ref. (*30*), we use a combination of a deterministic model and a stochastic diffusion model to generate the conditional distribution. We wish to sample the residual $r_{t+1}$ conditioned on the deterministic mean forecast $\mu_{t+1}$ and the previous mesoscale state $M_{t}$. We denote the conditioning vector by

$$y\;:=\left[\mu_{t+1};M_{t}\right]$$ (4)

where the ; indicates concatenation.

The diffusion training objective to be minimized can then be written as

$$E_{\sigma ,r,n}\left[\lambda \left(\sigma \right)∥D_{\phi }\left(r+n;y;\sigma \right)-r{∥}_{2}^{2}\right]$$ (5)

where the expectation is taken over noise levels σ sampled from the training noise distribution $p_{\text{train}}$, samples *r* from the target data distribution $p_{\text{data}}$, and Gaussian noise $n∼N\left(0,\sigma^{2}I\right)$. The function λ(σ) is an output scaling factor that, in conjunction with input scaling factors folded into the network $D_{\phi }$, maintains output signal magnitude within bounds as the noise level σ sampled during training and inference can vary greatly. The network formulation $D_{\phi }$ similarly includes preconditioning factors, which scale the input signal and noise magnitudes to make the training objective more suitable for encouraging better training dynamics using gradient descent. Because our work follows the prescription given in ref. (*42*), we direct the interested reader to follow the excellent exposition of design choice justifications in that reference. We select the DDPM++ U-Net architecture (*71*) as the backbone for the denoiser. The network has six encoder and decoder layers with a base embedding dimension of 128. During training, samples of Gaussian noise ($n∼N\left(0,\sigma^{2}I\right)$) are added to the training data samples to learn the denoising objective. The values of the SD σ are drawn from a distribution $p_{\text{train}}\left(\sigma \right)$, which was log-normal with mean −1.2 and SD 1.2. We remark that this choice of distribution of noise values was inherited from ref. (*42*) and is likely not optimal. Reference (*18*), for instance, chooses a different distribution of noise sampling during the training process. The specific form of the noise sampling distribution should be chosen so as to sufficiently sample values of noise that are relatively large and small compared to the variance of the individual channels in the training data samples. Reference (*42*) also takes into account the capacity of the network to learn the denoising objective at a range of values of σ while choosing a log-normal form for the noise sampling distribution. We train the network for a total of 450,000 training steps with batch size 64, corresponding to 29 million noised samples seen in training. The training pipeline completes in 120 hours on 64 NVIDIA H100 GPUs. At inference time, we initialize the random latent with Gaussian noise with SD σ = 800 and use the second order solver proposed in ref. (*42*) for 18 denoising steps to generate a sample given an input condition. In this configuration, an unoptimized inference implementation takes about 2 min to run a 12-hour forecast on a single A100 GPU and requires roughly 7 GB of GPU memory.

### Training data

#### Mesoscale state

Data for $M_{t}$ come from the operational archive of the US kilometer-scale forecasting model, the HRRR (*7*, *8*) on its native model grid. To mitigate nonstationarity from earlier HRRR versions, we only use data after July 2018 when HRRR v3 became operational. We use six dynamical variables (two horizontal wind components, temperature, geopotential height, pressure, and specific humidity) from a subset of 16 of the available 50 hybrid vertical levels, retaining complete vertical sampling of the atmospheric BL (see Table 1). Although we intended to train on the analysis state of the HRRR, i.e., at the time of its hourly data assimilation, we inadvertently used data 1 hour subsequent, early in its forecast phase. This could be viewed as helpful given that it takes time for the dynamical variables in the HRRR to adjust to what is previously assimilated to the extent that these adjustments are useful to include in the target.

**Table 1.** StormCast parameter set. Parameters from the ERA5 and HRRR dataset that are used for training the StormCast model.

ERA5		
Parameter	Pressure levels (hPa)	Height levels (m)
Zonal wind (u)	1000, 850, 500, 250	10
Meridional wind (v)	1000, 850, 500, 250	10
Geopotential height (z)	1000, 850, 500, 250	None
Temperature (t)	1000, 850, 500, 250	2
Humidity (q)	1000, 850, 500, 250	None
Total col. of water vapor (tcwv)	Integrated	–
Mean sea level press. (mslp)	Surface	–
Surface pressure (sp)	Surface	–
HRRR		
**Parameter**	**Hybrid model levels (index)**	**Height levels (m)**
Zonal wind (u)	1, 2, 3, 4, 5, 6, 7, 8, 9, 10, 11, 13, 15, 20, 25, 30	10
Meridional wind (v)	1, 2, 3, 4, 5, 6, 7, 8, 9, 10, 11, 13, 15, 20, 25, 30	10
Geopotential height (z)	1, 2, 3, 4, 5, 6, 7, 8, 9, 10, 11, 13, 15, 20, 25, 30	None
Temperature (t)	1, 2, 3, 4, 5, 6, 7, 8, 9, 10, 11, 13, 15, 20, 25, 30	2
Humidity (q)	1, 2, 3, 4, 5, 6, 7, 8, 9, 10, 11, 13, 15, 20, 25, 30	None
Pressure (p)	1, 2, 3, 4, 5, 6, 7, 8, 9, 10, 11, 13, 15, 20	None
Max. composite radar refl. (refc)	–	Integrated
Mean sea level press. (mslp)	–	Surface
Orography	–	Surface
Land/water mask	–	Surface

#### Synoptic state (training)

Data for $S_{t}$ come from the ERA5 (*27*) reanalysis. We use a sparse vertical sampling of six variables (two horizontal wind components, temperature, geopotential height, temperature, and specific humidity) across four pressure levels, each interpolated to the HRRR’s native horizontal and vertical grid.

#### Temporal resolution

We sample both the ERA5 and HRRR data at 1-hour intervals. That is, we create a paired dataset at the same valid time $t_{i}$ of HRRR and ERA5 snapshots ($M_{t_{i}},S_{t_{i}}$) with hourly time resolution.

#### Spatial resolution

The spatial resolution of the HRRR and StormCast is 3 km. We train StormCast on a spatial region over the Central US with spatial extent of 1536 km by 1920 km. Our vertical grid spacing is as fine as 125 m in the BL and as coarse as 500 m in the free troposphere. The horizontal resolution of the ERA5 conditioning data is ~28 km.

#### Data volume and train/test split

Approximately 3.5 years (30,660 independent samples) of training data are used, from July 2018 to December 2021, and therefore include both v3 and v4 of the operational HRRR analysis. Data for the year 2022 are held out for validation. Additional data during spring 2024 were used for testing in a forecast context as follows.

### Forecast experiment design

#### Evaluation period

A total of 135 StormCast forecasts are launched for the date range 8 May to 15 June 2024, four times daily at 0Z, 6Z, 12Z, and 18Z. The beginning of this time interval was constrained by the onset of our own collection of synoptic conditioning data $S_{t}$ from the real time NOAA Operational Model Archive and Distribution System (*72*), where it is cached at our desired hourly temporal frequency.

#### Initialization and conditioning

Each forecast used HRRR analysis states to initialize $M_{t}$ to permit the HRRR data assimilation scheme to constrain the initial mesoscale state. Synoptic conditioning was provided via hourly output from single deterministic 0.25°-resolution NCEP GFS global forecasts launched at common initial times.

#### Ensemble design

At each initialization, we generate a five-member ensemble forecast using StormCast. At each 1-hour forecast step, the diffusion stage samples a forecast state starting from Gaussian random noise with variation in this sampling generating an ensemble of forecasts. The ensembles are propagated forward autoregressively at each time step. Generating many more ensemble members is computationally cheap. However, probabilistic ensemble calibration requires further investigation. We expect to explore this aspect in future work.

#### Forecast validation

We compute forecast skill metrics over a small representative set of atmospheric variables—composite reflectivity, surface zonal and meridional wind at 10 m, surface temperature at 2 m, as well as specific humidity, temperature, and zonal wind at hybrid levels 5 and 10 (~700-m and 2.5-km altitude). For all variables except composite reflectivity, we treat the HRRR analysis at the verification time as the ground truth.

#### Forecast validation (radar)

For composite reflectivity, we consider the MRMS (*45*) observation at the verification time to be the ground truth. Recognizing that precipitation scoring requires nuance, we validate radar using the FSS (*43*, *44*). The FSS compares the number of grid cells with precipitation exceeding a given threshold in the forecast and in the verification through a neighborhood of fixed size surrounding every point in the domain. The FSS ranges between 0 and 1, with a perfect score of 1 meaning that the fraction of cells with precipitation above the threshold matched the verification through every neighborhood. This approach avoids the double penalty that would be incurred using a score, such as RMSE, if a correct precipitation intensity is slightly misplaced relative to the verification.

For a given threshold *t*, the true field $y_{\text{true}}$ and the predicted radar reflectivity fields $y_{\text{pred}}$ are discretized by

$$y_{\text{true}}^{\left(t\right)}\left(i,j\right)=\left\{\begin{array}{cc} 1, & \text{if}\;y_{\text{true}}\left(i,j\right)>t\\ 0, & \text{otherwise} \end{array}\right.$$

$$y_{\text{pred}}^{\left(t\right)}\left(i,j\right)=\left\{\begin{array}{cc} 1, & \text{if}\;y_{\text{pred}}\left(i,j\right)>t\\ 0, & \text{otherwise} \end{array}\right.$$

Next, we perform an average pooling operation over the target and predicted discretized fields with a specified neighborhood size *n*

$${\tilde{y}}_{\text{true}}^{\left(t,n\right)}\left(i,j\right)=\frac{1}{n^{2}}\sum_{k=0}^{n-1}\sum_{l=0}^{n-1}y_{\text{true}}^{\left(t\right)}\left(i+k,j+l\right)$$

$${\tilde{y}}_{\text{pred}}^{\left(t,n\right)}\left(i,j\right)=\frac{1}{n^{2}}\sum_{k=0}^{n-1}\sum_{l=0}^{n-1}y_{\text{pred}}^{\left(t\right)}\left(i+k,j+l\right)$$

The mean squared error in the predicted discretized and neighborhood pooled field is calculated as

$${\text{MSE}}^{\left(t,n\right)}=\frac{1}{N}\sum_{i,j}{\left[{\tilde{y}}_{\text{pred}}^{\left(t,n\right)}\left(i,j\right)-{\tilde{y}}_{\text{true}}^{\left(t,n\right)}(i,j)\right]}^{2}$$

where N is the total number of pixels in the discretized fields.

A reference mean squared error is computed for normalization

$${\text{MSE}}_{\text{ref}}^{\left(t,n\right)}=\frac{1}{N}\sum_{i,j}\left[{\tilde{y}}_{\text{pred}}^{\left(t,n\right)}{\left(i,j\right)}^{2}+{\tilde{y}}_{\text{true}}^{\left(t,n\right)}{\left(i,j\right)}^{2}\right]$$

Last, the FSS is computed by comparing the error ${\text{MSE}}_{n}$ to the reference error ${\text{MSE}}_{n}^{\text{ref}}$ as follows

$${\text{FSS}}^{\left(t,n\right)}=1-\frac{{\text{MSE}}^{\left(t,n\right)}}{{\text{MSE}}_{\text{ref}}^{\left(t,n\right)}}$$

An FSS of 1 indicates a perfect match between the predicted and true fields, whereas an FSS of 0 corresponds to the error equaling the reference error.

We indicate the spatial scale of the FSS as the size of the neighborhood used for the pooling operation in the FSS. Thus, a neighborhood size of *n* = 5 indicates pooling over a neighborhood of 5 by 5 pixels or equivalently 15 km by 15 km given the 3-km spatial resolution of the grid.

#### Forecast validation (radar and ensemble)

To compare our StormCast ensemble forecast of radar reflectivity with the single deterministic HRRR forecast, we compute the PMM (*73*, *74*) of the FSS. The PMM has been shown in a prior study (*75*) to be a more skillful estimate of a precipitation forecast from a CAM than individual ensemble members of the CAM as well as the plain ensemble average. The PMM is obtained as follows. We first compute an ordinary ensemble mean of the forecast and sort all values in the ensemble mean in ascending order. Each gridpoint in the domain is assigned a rank based on its index in the sorted order. Next, all values in the *n* ensemble members are flattened and sorted in ascending order and every *n*th value is retained. The retained values are assigned to gridpoints in the domain according to the rank of the gridpoint as computed from the ensemble mean sorting process.

#### Intercomparing ensemble and deterministic results

Unfortunately, corresponding ensemble forecasts from numerical models such as the HRRR are computationally expensive and were not available to us in the operational archive. Because this means we do not have a fair physics-based CAM ensemble baseline to compare against StormCast ensemble forecasts, the appropriate interpretation of our comparisons of StormCast to the HRRR is of the potential advantages of an ensemble generative ML approach. Our evaluation period had a small overlap of 15 days with the NOAA HWT SFE (https://hwt.nssl.noaa.gov/sfe/2024/). We obtained a small set of five-member ensemble forecasts from the NSF-NCAR MPAS ensemble forecasts (*56*, *57*) run as a part of this experiment. The MPAS ensembles were not initialized with a radar-assimilating analysis like the HRRR and StormCast forecasts were, hence the forecasts are not directly comparable. Even so, we include a cursory comparison in Fig. 8 with the intention of expanding this analysis in future work. We note that both HRRR and StormCast significantly outperform the MPAS forecasts in the first 6 hours due to the radar initialization. We also create an approximate HRRR ensemble using time-lagged ordinary HRRR forecasts, a technique commonly used in weather forecasting first introduced in ref. (*46*). The ensemble comprises a control forecast initialized at 00:00 UTC along with lagged forecasts initialized 1, 2, 3, and 4 hours earlier. At each verification time, the ensemble forecast consists of these lagged forecasts, each adjusted to the appropriate forecast lead time corresponding to the verification time. See the Supplementary Materials for further illustration how a lagged ensemble forecast is created by combining forecasts initialized at different times using the HRRR model.

## Supplementary Materials

### This PDF file includes:

Supplementary Text Figs. S1 to S21 References
