# Supplementary material for: Kilometer-scale convection-allowing model emulation using generative diffusion modeling
Source: Sci Adv. 2026 Jan 30;12(5):eadv0423. doi: 10.1126/sciadv.adv0423 (PMC12857735; doi:10.1126/sciadv.adv0423)
Supplement: Supplementary file 1 — Supplementary Text Figs. S1 to S21 References [file sciadv.adv0423_sm.pdf]

Science Advances
AAAS

# Supplementary Materials for

## Kilometer-scale convection-allowing model emulation using generative diffusion modeling

Jaideep Pathak *et al.*

Corresponding author: Jaideep Pathak, jpathak@nvidia.com; Yair Cohen, yair.chn@gmail.com; Piyush Garg, Piyush.Garg@rwe.com; Peter Harrington, pharrington@nvidia.com; Michael Pritchard, mpritchard@nvidia.com

*Sci. Adv.* **12**, eadv0423 (2026)
DOI: 10.1126/sciadv.adv0423

**This PDF file includes:**

Supplementary Text
Figs. S1 to S21
References

# Supplementary Text

## Qualitative assessment of convective dynamics through cold pool visualizations

Atmospheric cold pools formed in the vicinity of convection are dense masses of air cooler than the surrounding environment which descend from cloud bases and spread out along the ground (*76*). Cold pools are created when precipitation evaporates as it falls through the drier air underneath the convective storm (*77*). This evaporation cools the surrounding air, making it denser than the environment, leading to the formation of a density current near the surface. This cooler air spreads outward horizontally, forming a roughly circular/oval shape. Typical characteristics of cold pools are that their in-pool temperature is typically $1.5°C$-$10°C$ cooler than the environmental air (*78*, *79*, *80*). In addition, they are accompanied by strong, gusty winds at the leading edge, forming a gust-front signature near the surface (*79*). Their average diameter can range from a few kilometers (from small convective cores) to over 100 km (related to MCS or squall line thunderstorms) (*81*). Cold pools play an integral role in controlling thunderstorm dynamics and can trigger new convection by lifting warm, moist air at their boundaries (*78*). Although they are important in controlling the convective dynamics in an unstable environment, these mesoscale features are not well represented in current weather models (*82*).

The presence of cold pools in StormCast simulations is a useful test of its ability to learn an implicit representation of the relationship between precipitation production and evaporative cooling that is the source of negative buoyancy for cold pools. The co-development of thermal fronts and surface wind anomalies by cold pools is another test of multi-variate physical consistency in the model. To evaluate this, we identify cold pool related gust front signatures in the 10m near-surface wind fields (u10m and v10m) and air temperature at 125 m above the Earth's surface. We calculate horizontal wind and temperature gradients (*83*) using finite differences, followed by edge enhancement using Sobel filter (*84*). Figure S1 displays composite reflectivity contours at 40 and 50 dBZ with scalar wind and temperature gradient fields for both the target (top rows) and StormCast (bottom rows). These fields are depicted at 4 hours after initialization time to determine if StormCast has learned the cold pool representation or is merely deriving it from initial conditions.

Cold pools are known to have a distinct signature in temperature and wind fields (*80*, *83*) as the gust fronts resulting from rain evaporation exhibit stronger winds and lower temperatures compared

to the surrounding air mass. Figure S1 depicts distinct arc-like features of maximized gradient in both fields surrounding convection in the target HRRR data between Illinois and Missouri. Likewise, StormCast 4-hour forecasts contain arc-like structures surrounding portions of its most intense generated radar reflectivity feature over Missouri. Another gust front signature between Illinois and Indiana is clearly visible in both target and StormCast gradient fields. As in the target data, some co-location in both the temperature and horizontal wind gradient maxima are apparent in StormCast.

## Additional spectrum analysis

A more extensive analysis of the spectra and distribution is shown in figure S2, for surface winds as well as some other core variables. These reinforce the conclusion in the main text as StormCast is able to capture the spectra of many channels, correctly representing it even at the smallest scales.

We further analyze the lead time-dependent error in the spectra relative to the target data. This relative error in the spectra, $\epsilon_r(f)$ is defined at a given frequency $f$, according to:

$$\epsilon_r(f) = \frac{m(f)}{t(f)} - 1 \tag{S1}$$

where $m(f)$ and $t(f)$ respectively denote the spectral density of the model and target at a frequency $f$. This dimensionless ratio effectively highlights the differences in spectra generated by StormCast across various lead times, which are not readily apparent when plotting dimensional spectra. The relative error in StormCast's spectra is primarily localized at smaller scales in most variables. StormCast's spectral error consistently increases with lead time, with the highest values observed at 12-hour lead times (see figure S3). Notably, for the first 6 hours—during which predictability of convective-scale motions can be expected—StormCast maintains a realistic spectrum across a range of channels.

The spectra and distributions for lead times, 1, 3, 6 and 12 hours are shown in Figs. S4, S5, S6, S7 respectively.

## Lagged Ensemble HRRR Baseline

Figure S8 illustrates the methodology of creating a lagged ensemble forecast using HRRR forecasts.

Figure S9 extends the analysis presented in Fig.4 by evaluating the Fractions Skill Score (FSS) of composite radar reflectivity forecasts at additional spatial scales. FigureS10 directly compares

the accuracy of composite radar reflectivity forecasts from the Probability Matched Mean (PMM) of a lagged HRRR ensemble to those from a single ordinary HRRR forecast. Finally, Figure S11 contrasts radar reflectivity forecasts generated by the StormCast Ensemble PMM against forecasts from an individual StormCast control member.

At almost all forecast lead times, spatial scales and models considered, the forecast skill of the PMM of an ensemble forecast exceeds that of an individual ensemble member from that ensemble. This is true in the case of StormCast diffusion generated ensemble forecasts as well as approximate ensembles generated using forecast lags from ordinary HRRR forecasts. This finding provides motivation for further research on the use of generative models for creating mesoscale ensemble forecasts.

### Additional Vertical Sections

We complement the analysis in Figure 7 with two additional figures from different meteorological scenarios. Figure S12 illustrates the 6th hour of a simulated nocturnal Mesoscale Convective System (MCS) over Missouri. The convective system is discernible in both the planar view of radar reflectivity (top row) and the vertical-zonal slice of moist enthalpy (second row). It is encouraging that 6 hours into a forecast StormCast produces a plausible MCS reflectivity morphology including a core of intense convection embedded within a larger region of lower radar reflectivity reminiscent of a leading virga anvil. The eastward-moving cloud system is characterized by a positive enthalpy anomaly concentrated in the eastern section of its cloud area, contrasting sharply with a negative enthalpy anomaly in its wake, consistent with a trailing mesoscale downdraft. Realistic generated multivariate updraft morphology is apparent at two longitudes of peak radar reflectivity ($-89.5°E$ and $-87°E$) where plumes of enthalpy extending into the boundary layer co-locate with net BL convergence and peaks in radar reflectivity . Figure S13 depicts the 6th hour of a simulated squall line over Texas, representing a clear case of a synoptically driven system. Again, the diffusion component of StormCast is found to produce spatially co-located km-scale anomalies in radar reflectivity, net BL convergence, and penetrative enthalpy plumes.

These additional cases demonstrate the model's capability to capture diverse convective structures and highlight the varying degrees of improvement offered by the diffusion model across different meteorological conditions. The analysis underscores the model's strength in enhancing fine-scale

features, particularly in radar reflectivity fields, while maintaining consistency with larger-scale structures present in the conditioning data.

### Ensemble Calibration

We perform a preliminary analysis of the calibration of the small ensemble forecasts generated by StormCast. We compute the ratio of the ensemble spread and the ensemble mean RMSE – called the spread-error ratio – as a function of the lead time:

$$\mathrm{SER} = \frac{\mathrm{StdDev}\left(\{x_{ens}\}_{i=1}^{ens}\right)}{\mathrm{RMSE}\left(\langle x_i \rangle_{i=1}^{ens}, x_{target}\right)} \times \sqrt{\frac{ens+1}{ens}} \tag{S2}$$

where $\{x_{ens}\}_{i=1}^{ens}$ is an ensemble forecast with $ens$ denoting the number of ensemble members, $\langle \cdot \rangle$ indicates an ensemble mean and $x_{target}$ denotes the ground truth or target verification data.

A perfectly calibrated forecast will have a constant spread-error ratio of 1 indicating that the spread in the ensemble members is perfectly indicative of the uncertainty in the forecast.

Our results shown in Fig. S14 indicate an under-dispersive ensemble. Calibration of ML forecasts is an active direction of research in the medium-range weather domain (*19*, *20*, *85*) and we expect similar efforts will be required to obtain properly calibrated forecasts of mesoscale weather. Future directions for improving the calibration of the ensemble could include using a GEFS ensemble as synoptic scale conditioning, incorporating initial condition uncertainty in the initialization of StormCast, improved diffusion sampling techniques for promoting ensemble diversity in addition to improving the forecast skill of StormCast.

### Verifying Composite Reflectivity Forecasts Against HRRR Analysis

In Sec.  we compare composite radar reflectivity forecasts made by StormCast and HRRR to the observed composite radar reflectivity from the MRMS product. In other words, the MRMS observations are considered the ground truth against which forecasts are verified. Typically, best practice dictates that forecasts be directly verified against observations where possible in order to avoid errors and biases introduced by the data-assimilation procedure that creates analysis states. Even so, a second option would be to consider the composite reflectivity analysis produced by the HRRR model to be the ground truth. In Figure S15, we compare the two options. We compute FSS

at grid scale and three different thresholds for the StormCast ensemble PMM and HRRR composite radar reflectivity forecasts. For each forecast, we compare against MRMS and HRRR analysis as the ground truth. The change of verification ground truth affects the HRRR and StormCast forecasts uniformly. At a 20dBZ threshold, the forecasts appear more skilled when compared against HRRR analysis than when compared against MRMS observations. However at 30dBZ and 40dBZ thresholds, the change of verification data appears to have negligible effects on both the HRRR and StormCast FSS.

### Additional forecast case studies

Figures S16-S21 show additional forecast visualizations from StormCast

# Figures and Figure Captions

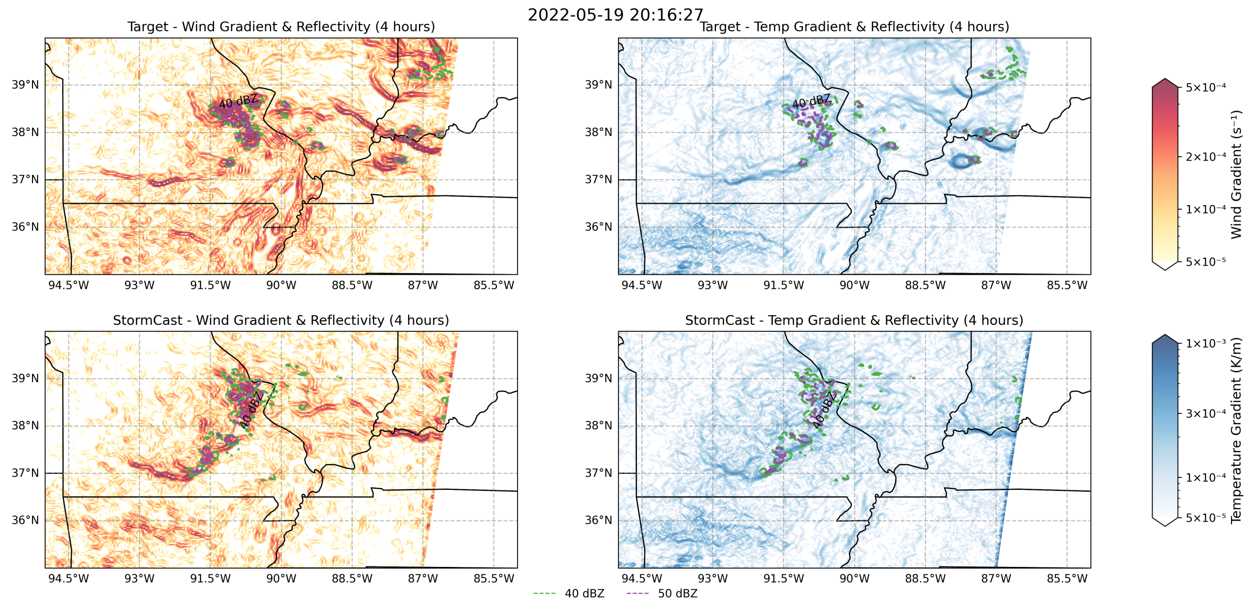


**Figure S1**: **Fine-scale horizontal wind gradients** Representative fine-scale features of the horizontal gradients of 10m horizontal wind (left column) and 125m air temperature gradient (right column) for target (top rows) and StormCast 4-hour forecasts (bottom rows), suggestive of cold pool related gust fronts. This case was initialized on 2022-05-19 17:30:00 UTC. Composite radar reflectivity contours at 40 and 50 dBZ are overlain in green and purple.

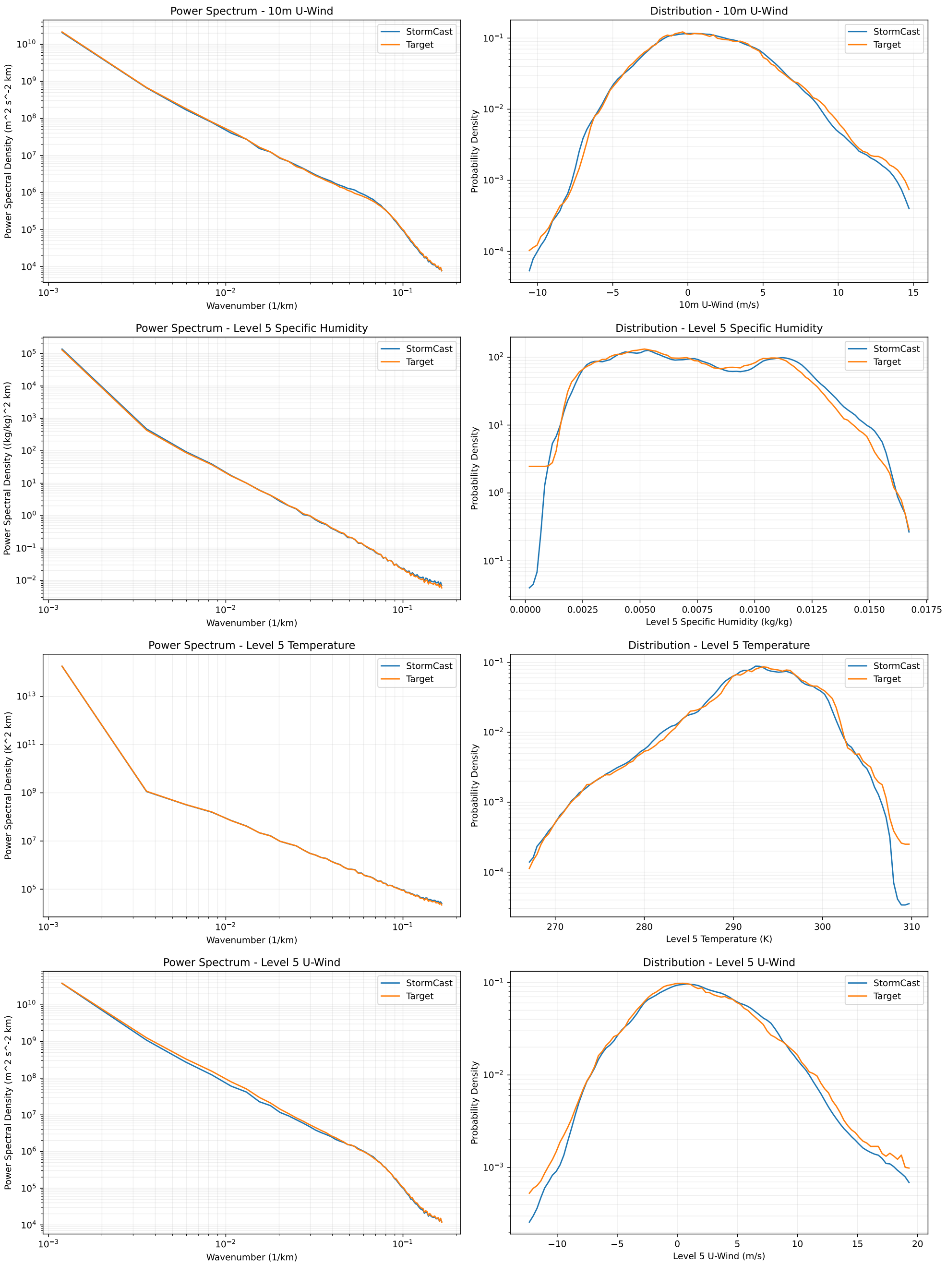


**Figure S2**: **Power spectra and distribution of select representative channels.** As figure 6 but for 10m U wind (top row), Level 5 Specific Humidity (second row), Level 5 Temperature (third row), Level 5 U wind (Fourth Row).

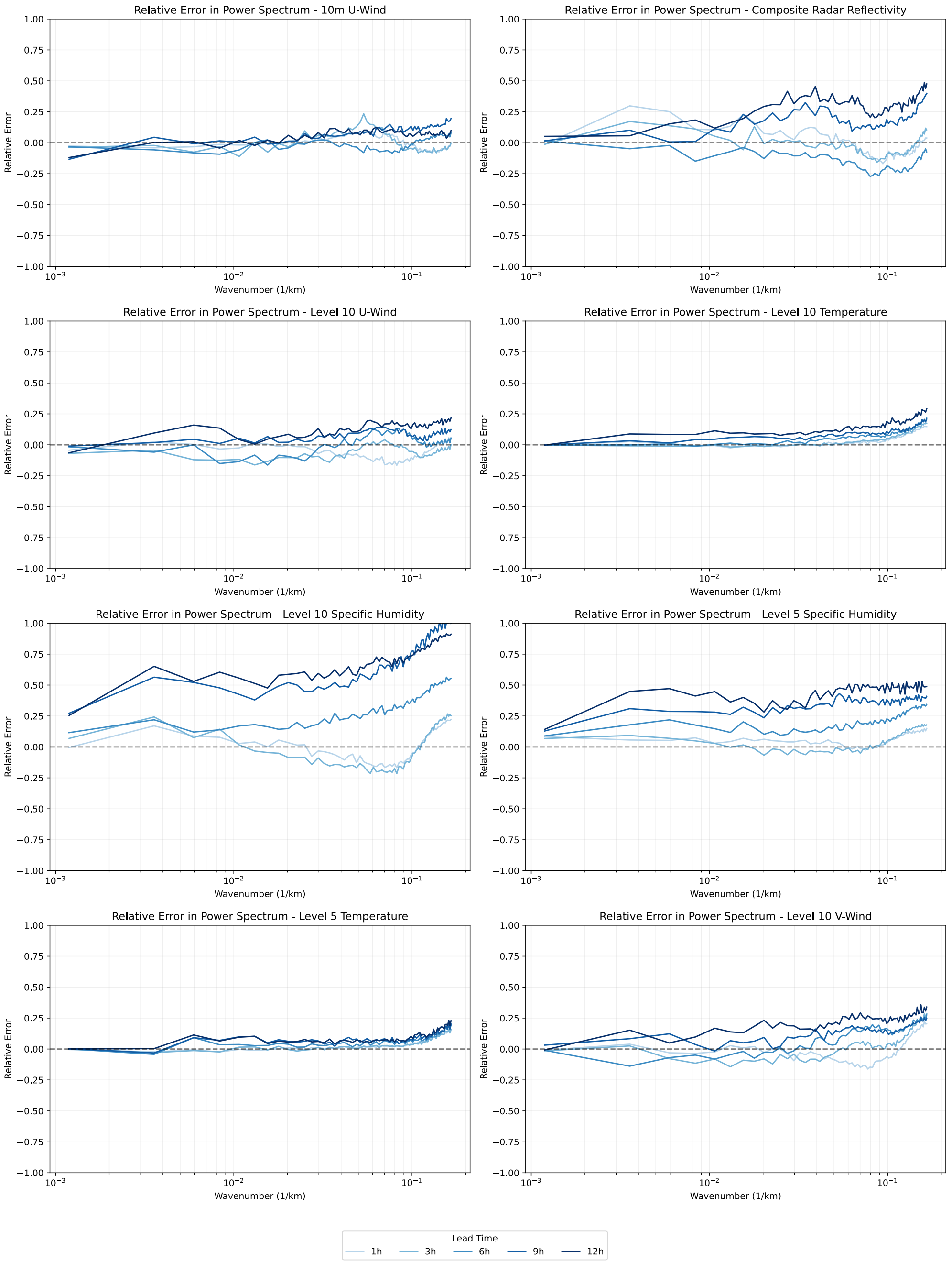


**Figure S3**: **Relative error in spectra of atmospheric variables as a function of lead time.** The relative error of the spectra defined in Eq. S1computed for various channels as a function of lead time (see legend).

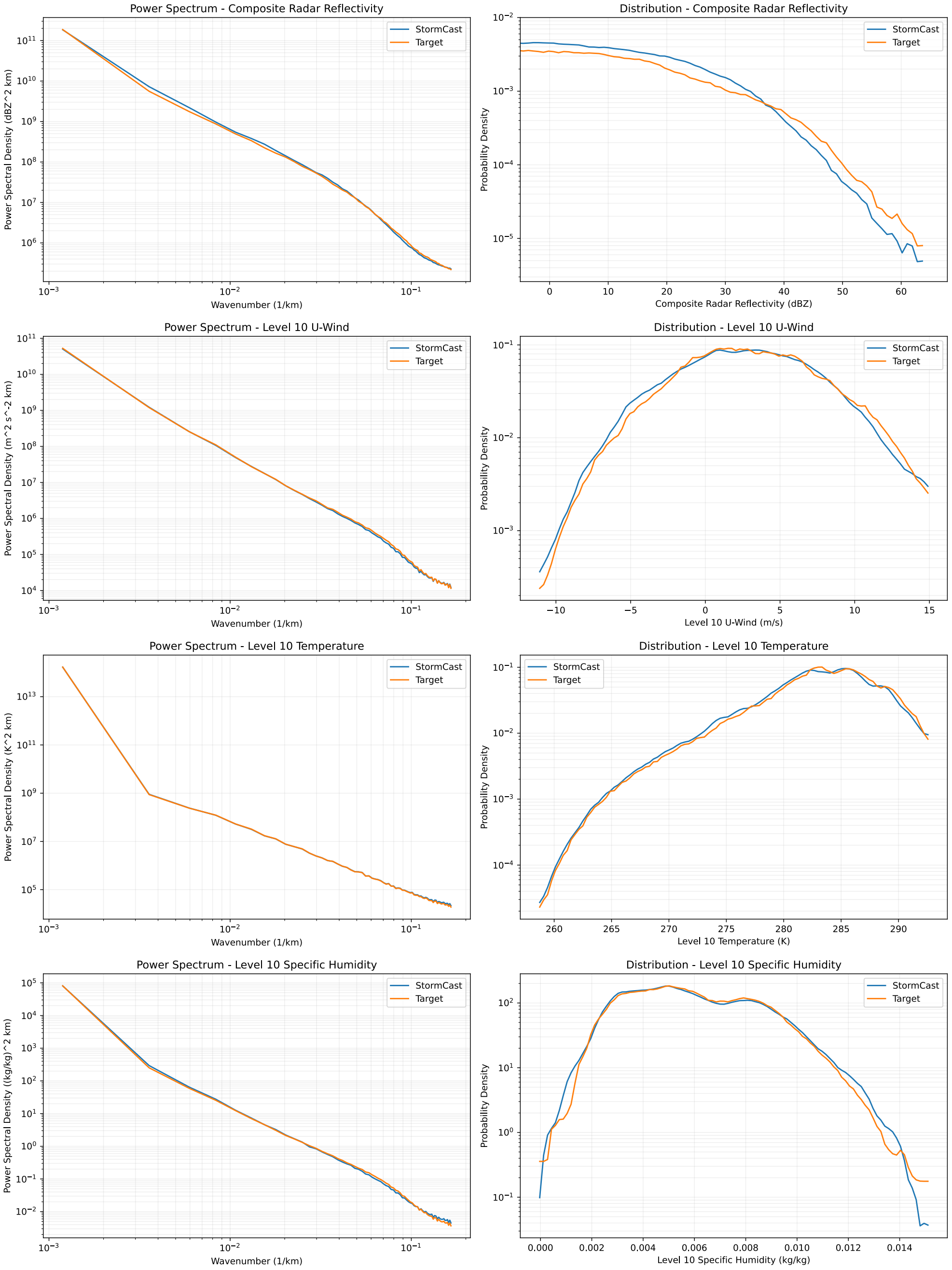


**Figure S4**: **Spectra and distributions of select variables from StormCast at 1h lead time** As figure 6 but for lead time 1h.

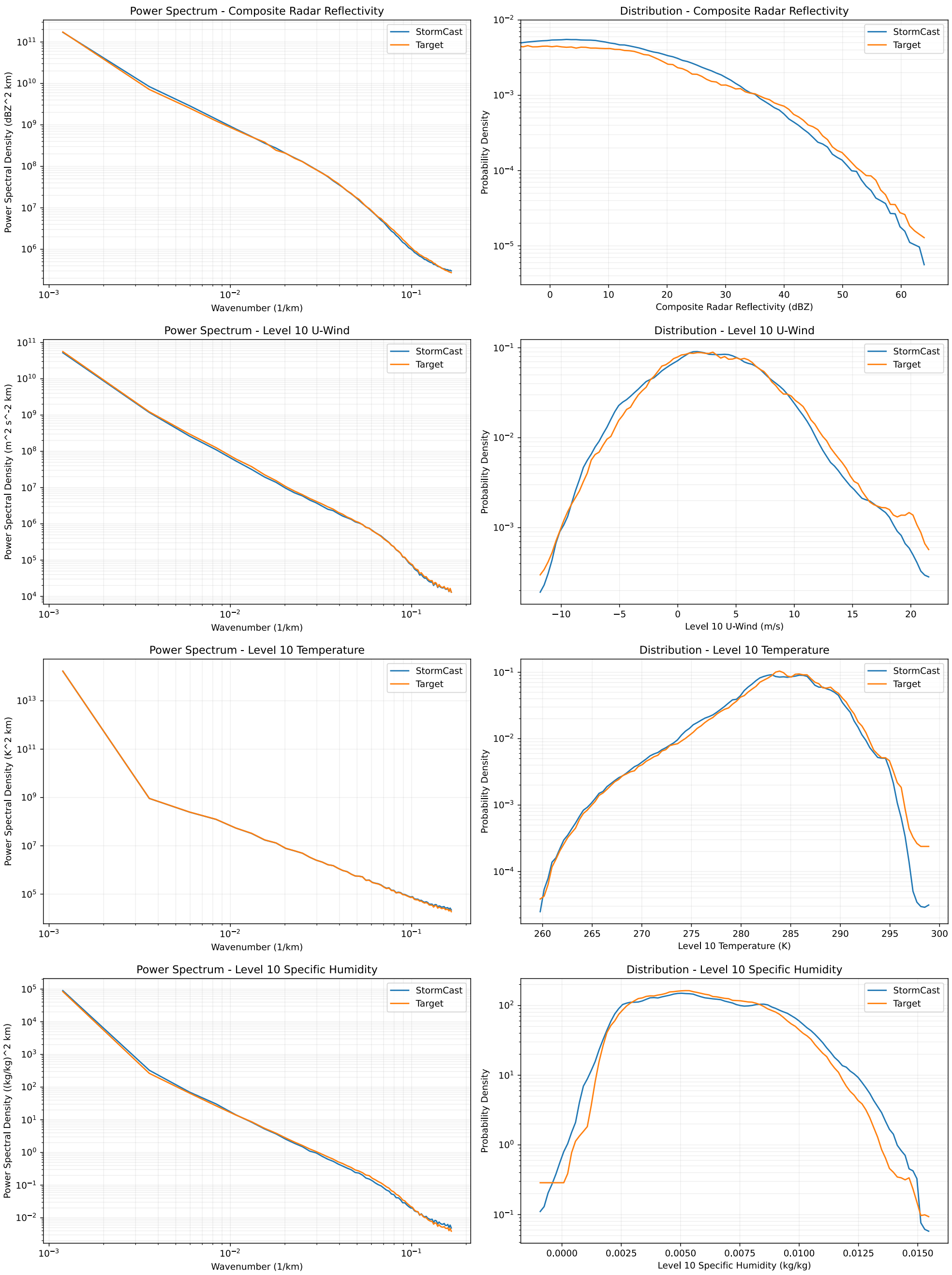


**Figure S5**: **Spectra and distributions of select variables from StormCast at 3h lead time** As figure 6 but for lead time 3h.

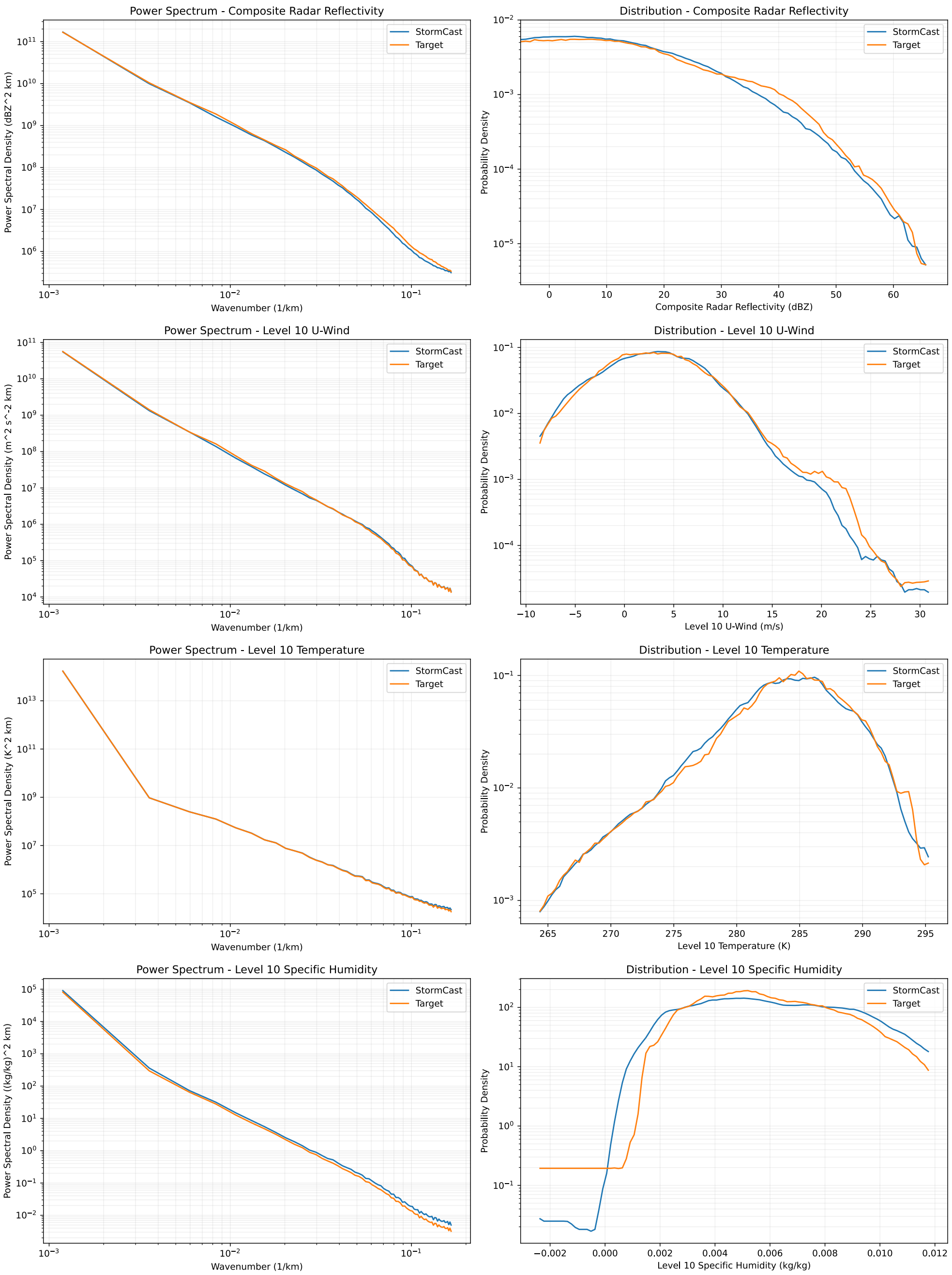


**Figure S6**: **Spectra and distributions of select variables from StormCast at 6h lead time**. As figure 6 but for lead time 6h.

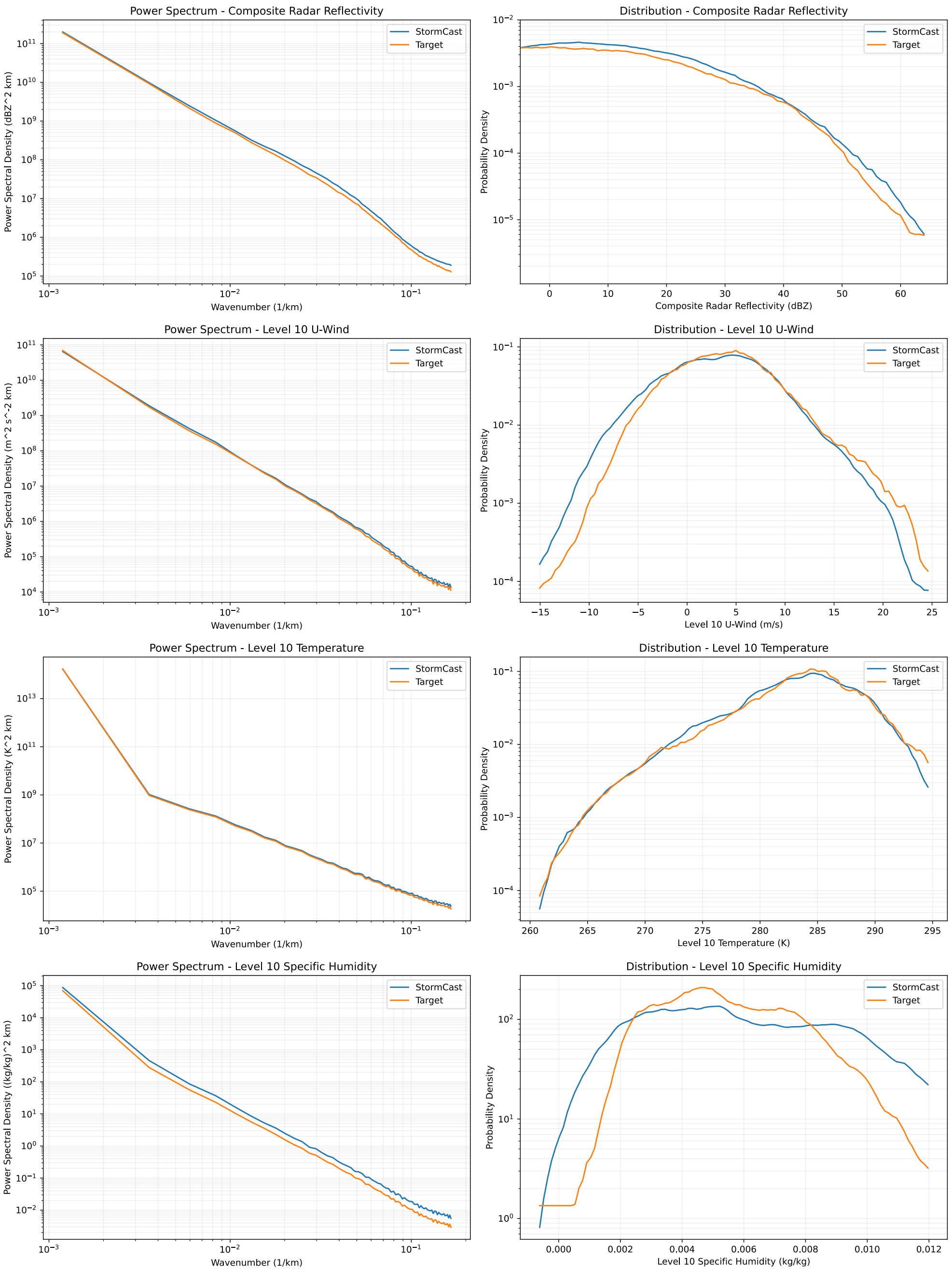


**Figure S7**: **Spectra and distributions of select variables from StormCast at 12h lead time.** As figure 6 but for lead time 12h.

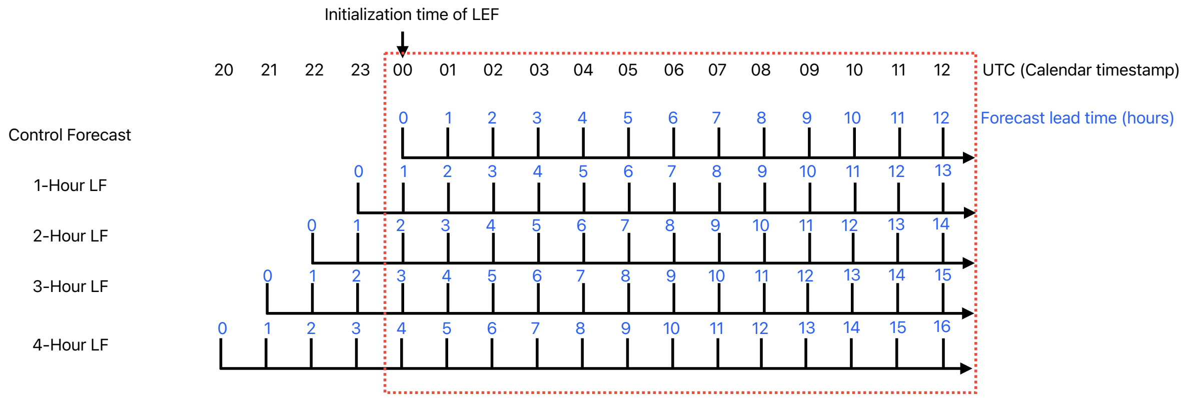


**Figure S8**: **Illustration of a time-lagged ensemble generated from HRRR forecasts.** The Schematic show a lagged ensemble forecast with the control forecast initialized at 00 UTC and the inclusion of lagged forecasts initialized at 1-hour intervals (1–4 hours prior). Ensemble members are selected by matching their respective forecast lead times to the designated verification time.

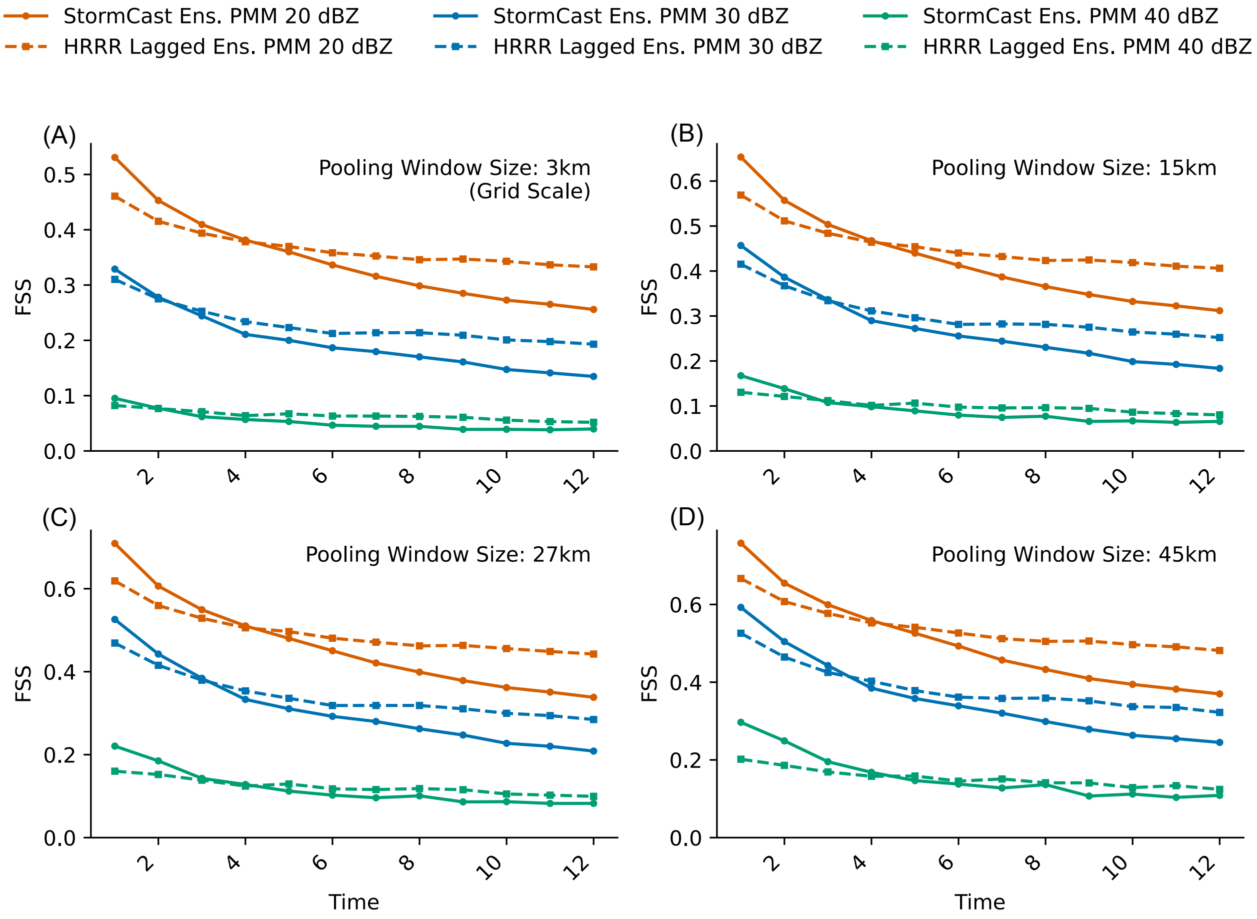


**Figure S9**: **Quantitative comparison of composite radar reflectivity forecast skill between HRRR time-lagged ensembles and StormCast ensembles** Fractions Skill Score (FSS) comparison for composite radar reflectivity between forecasts generated by the PMM of the HRRR Lagged Ensemble and the Ensemble PMM across varying spatial scales – 3 km (panel A), 15 km (panel B), 27 km (panel C), and 45 km (panel D).

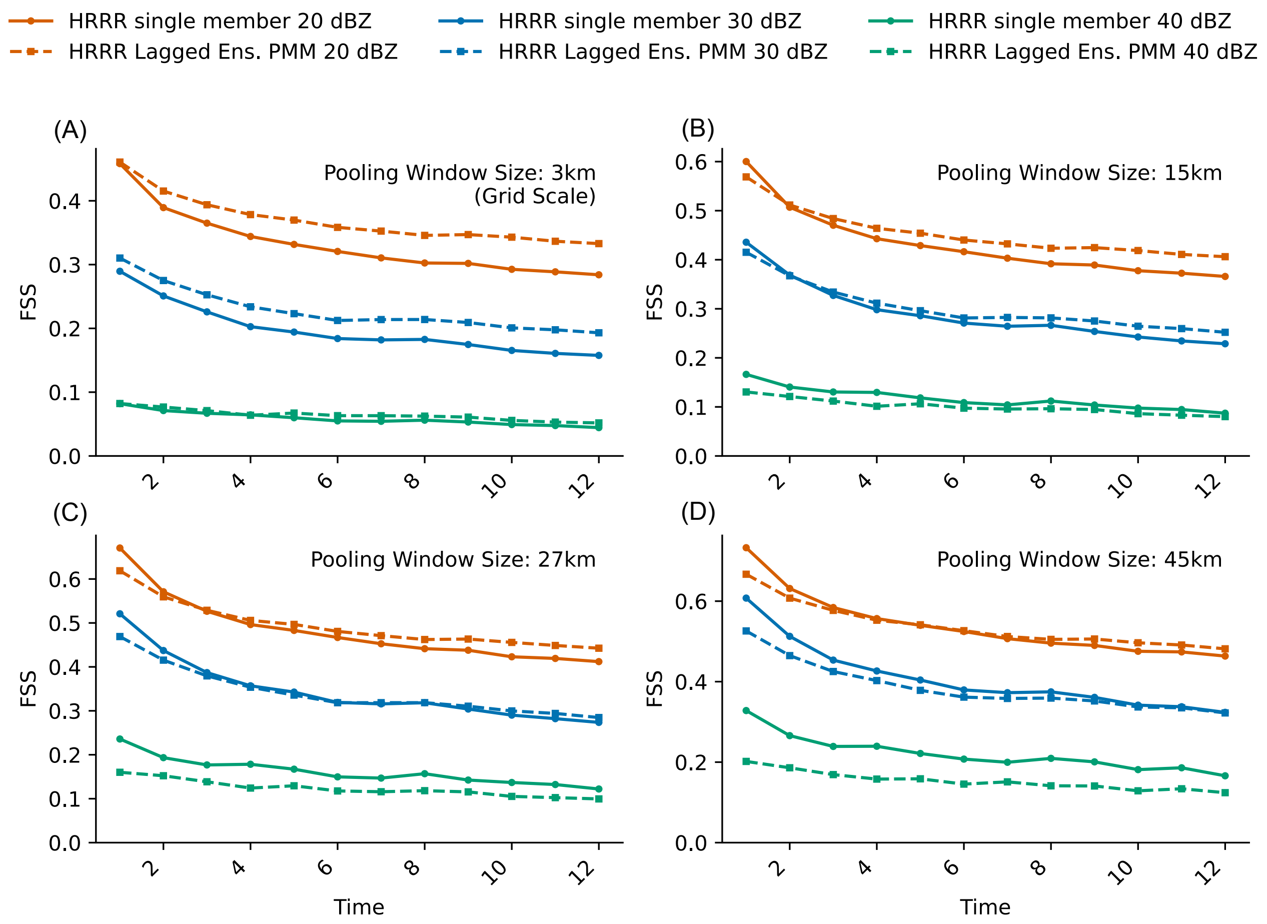


**Figure S10**: **Quantitative comparison of composite radar reflectivity forecast skill between HRRR time-lagged ensembles and HRRR single member forecasts.** Fractions Skill Score (FSS) evaluation of composite radar reflectivity comparing forecasts from the HRRR Lagged Ensemble PMM and a standard (ordinary) HRRR forecast at spatial scales of 3 km (panel A), 15 km (panel B), 27 km (panel C), and 45 km (panel D).

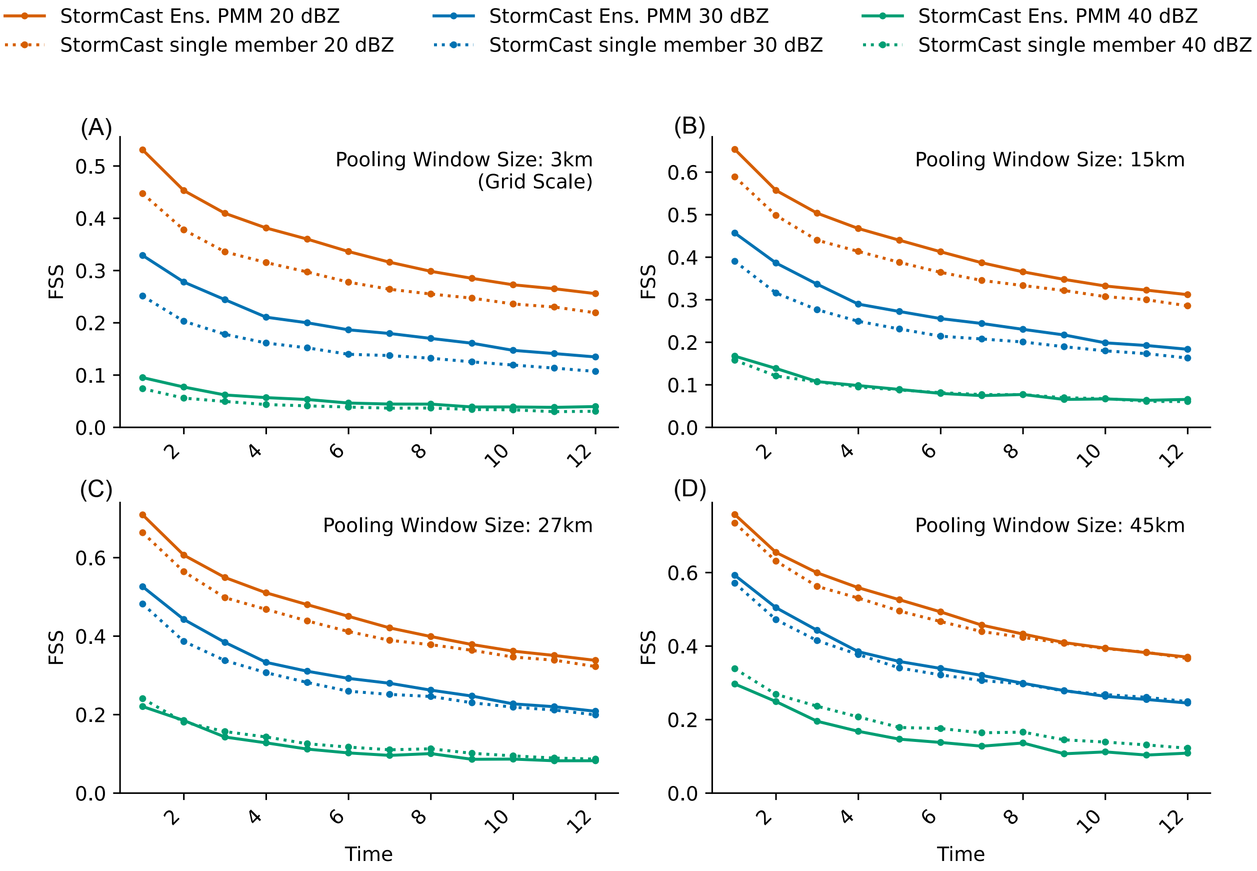


**Figure S11**: **Quantitative comparison of composite radar reflectivity forecast skill between StormCast ensembles and StormCast single member forecasts.** Fractions Skill Score (FSS) comparison between composite radar reflectivity forecasts made by the StormCast Ensemble PMM and a single-member StormCast control forecast at spatial scales of 3 km (Panel A), 15 km (Panel B), 27 km (Panel C), and 45 km (Panel D).

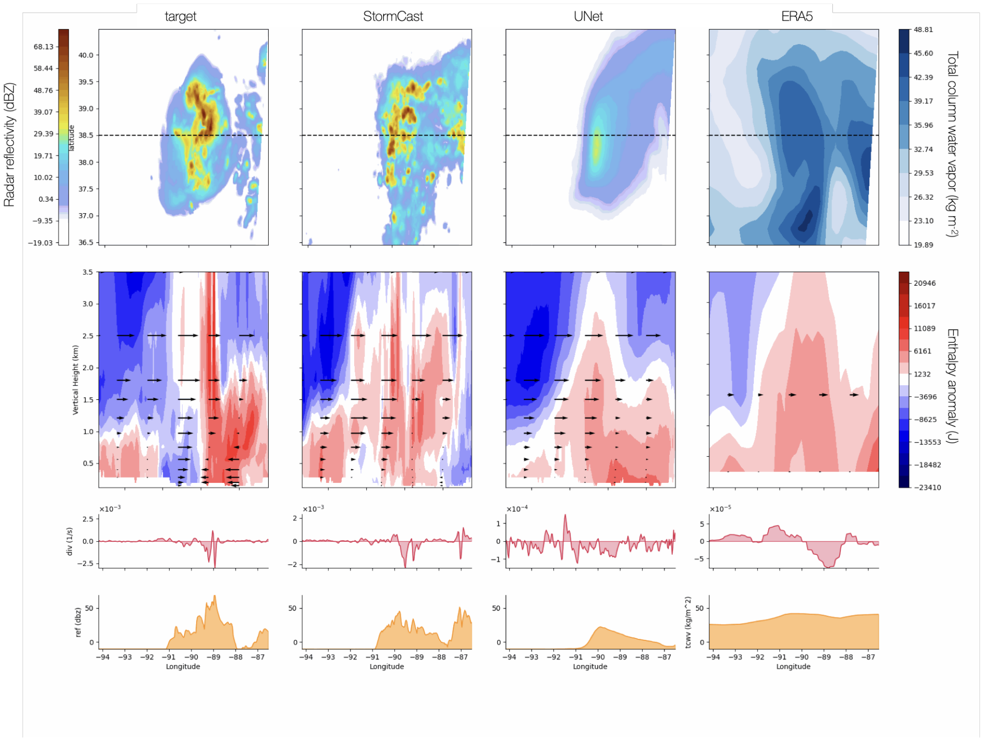


**Figure S12**: **Qualitative analysis of a StormCast Forecast.** Examining a nocturnal MCS in Missouri on 2022-05-19 23:00 UTC at a lead time of 6h into the simulation. The figure format follows that of 7. Additionally the third column (titled UNet) shows the output of StormCast with only the regression model applied and excluding the diffusion model. The fourth column shows the total column water vapor from ERA5 data as a visual guide for synoptic-scale conditions (top row) with the enthalpy anomaly computed from ERA5 data (second row), BL divergence (third row), and a section of total column water vapor (bottom row) at the indicated latitude line.

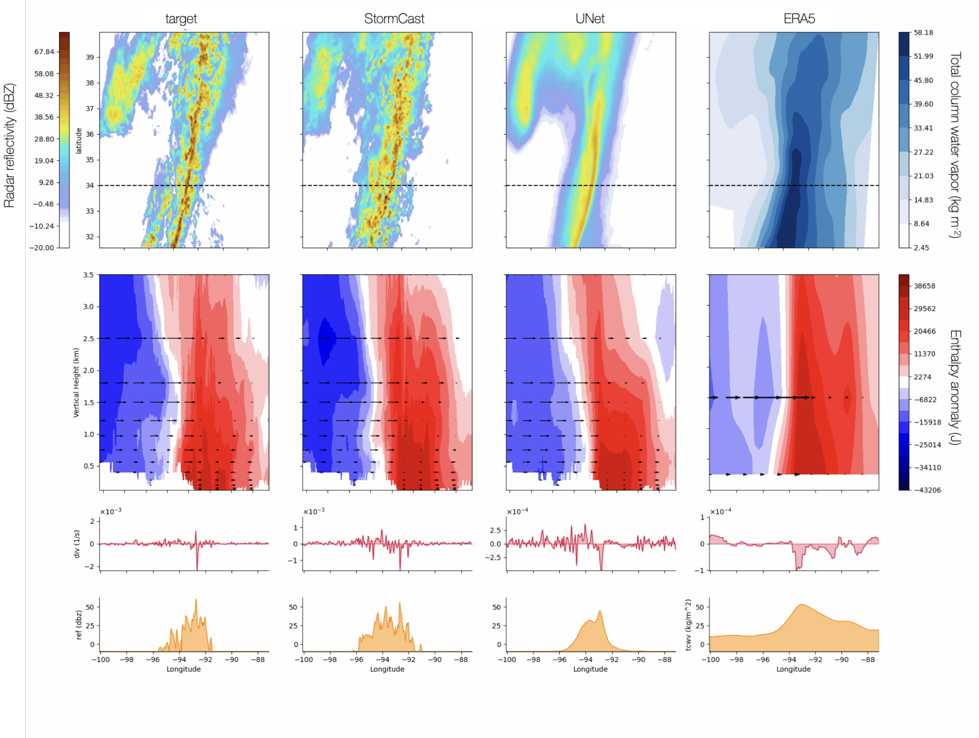


**Figure S13**: **Qualitative analysis of a StormCast Forecast.** Examining a squall-line passing in Texas on 2022-11-05 03:00 UTC at a lead time of 6h into the simulation. The figure format follows that of 7. Additionally the third column (titled UNet) shows the output of StormCast with only the regression model applied and excluding the diffusion model. The fourth column shows the total column water vapor from ERA5 data as a visual guide for synoptic-scale conditions (top row) with the enthalpy anomaly (second row), BL divergence (third row), and a section of total column water vapor (bottom row) at the indicated latitude line.

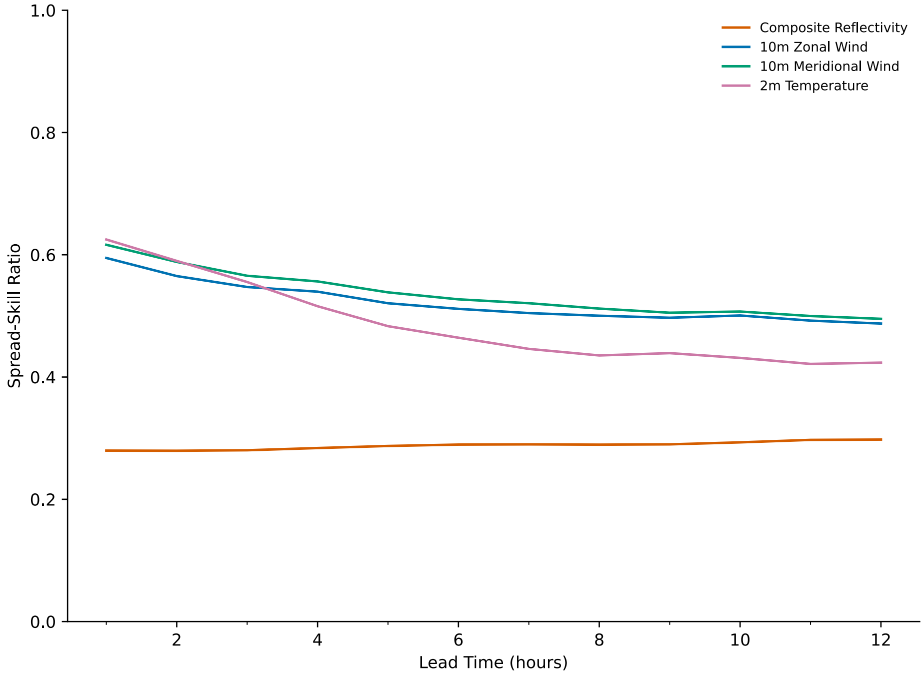


**Figure S14**: **Spread-error ratio of StormCast ensemble forecasts**. The spread-error ratio of a 5-member ensemble forecast made by StormCast indicating an underdispersive ensemble generated by StormCast.

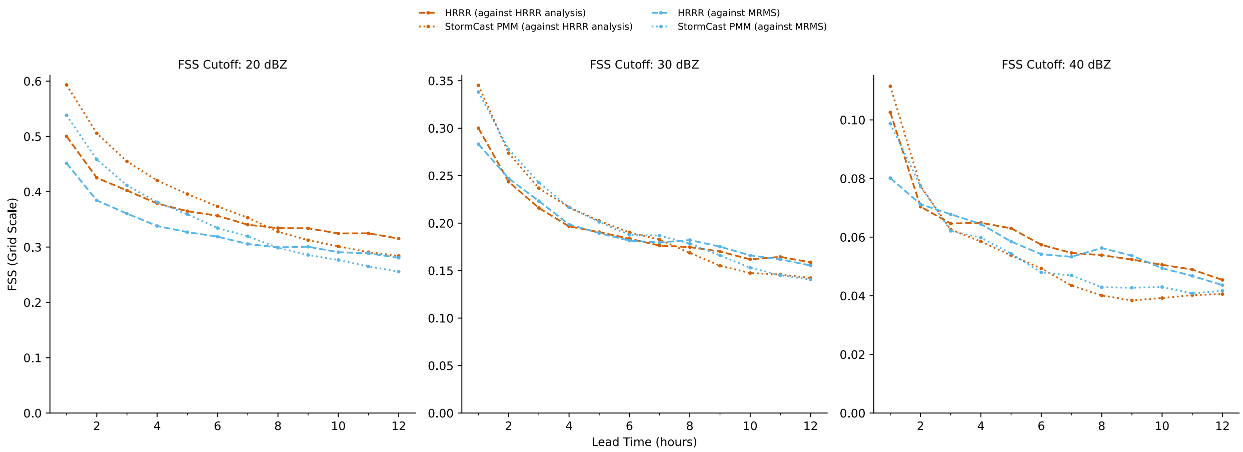


**Figure S15**: **Effect of verification data source for composite reflectivity forecasts**. Comparison of FSS for composite radar reflectivity forecasts from StormCast ensemble Probability Matched Mean (dotted lines) and HRRR (dashed lines), verified using two different reference datasets as ground truth: MRMS observations (blue color) and HRRR analysis fields (orange color). The FSS is evaluated at grid scale for reflectivity thresholds of 20 dBZ, 30 dBZ, and 40 dBZ. At the 20 dBZ threshold, forecasts exhibit higher skill when verified against HRRR analysis compared to MRMS observations. For higher reflectivity thresholds (30 dBZ and 40 dBZ), the choice of verification dataset has negligible effects on forecast skill scores.

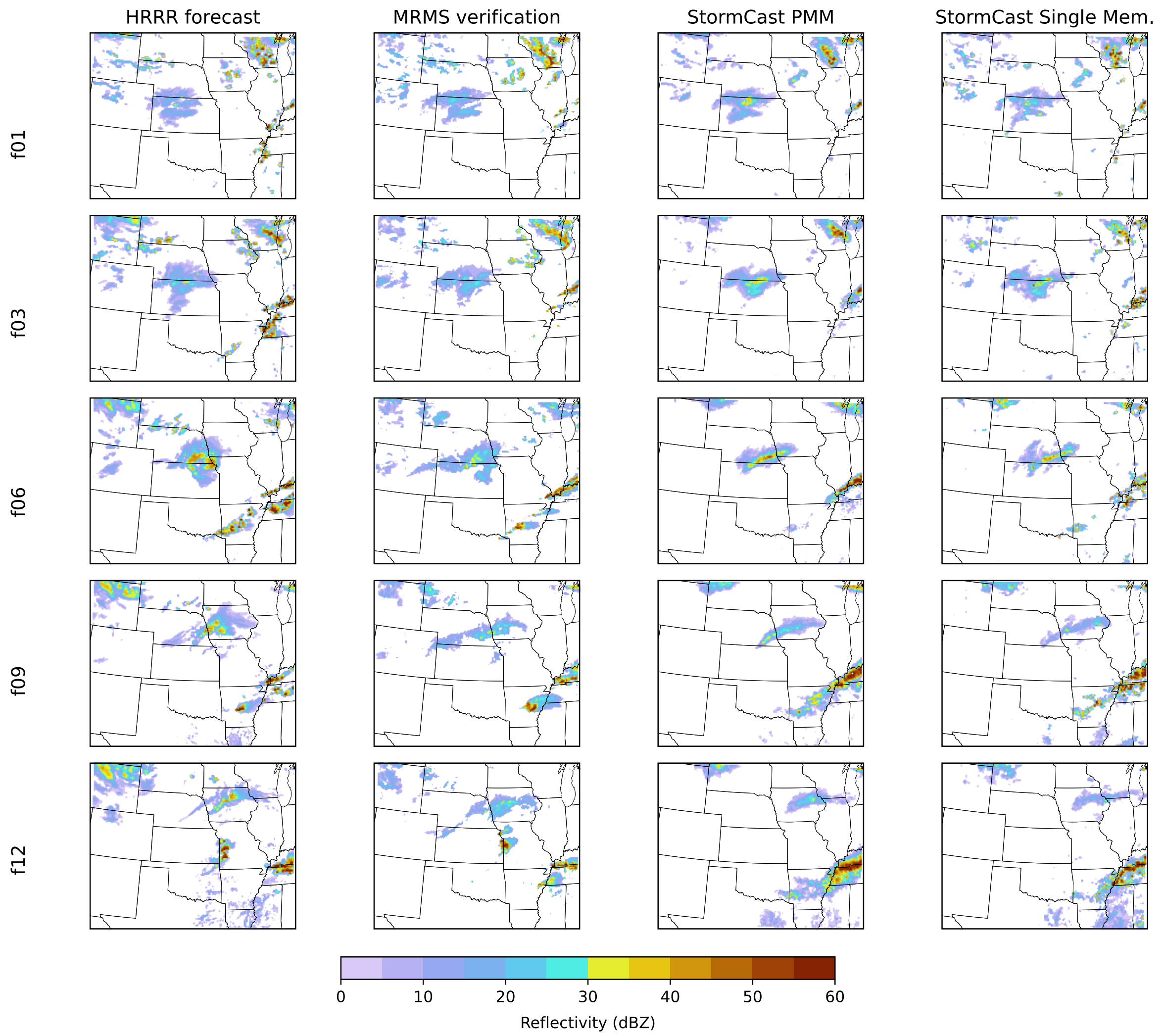


**Figure S16**: **Example forecast visualization from StormCast compared against MRMS verification and a corresponding HRRR forecast**. 2024-05-08 00:00:00 initialization

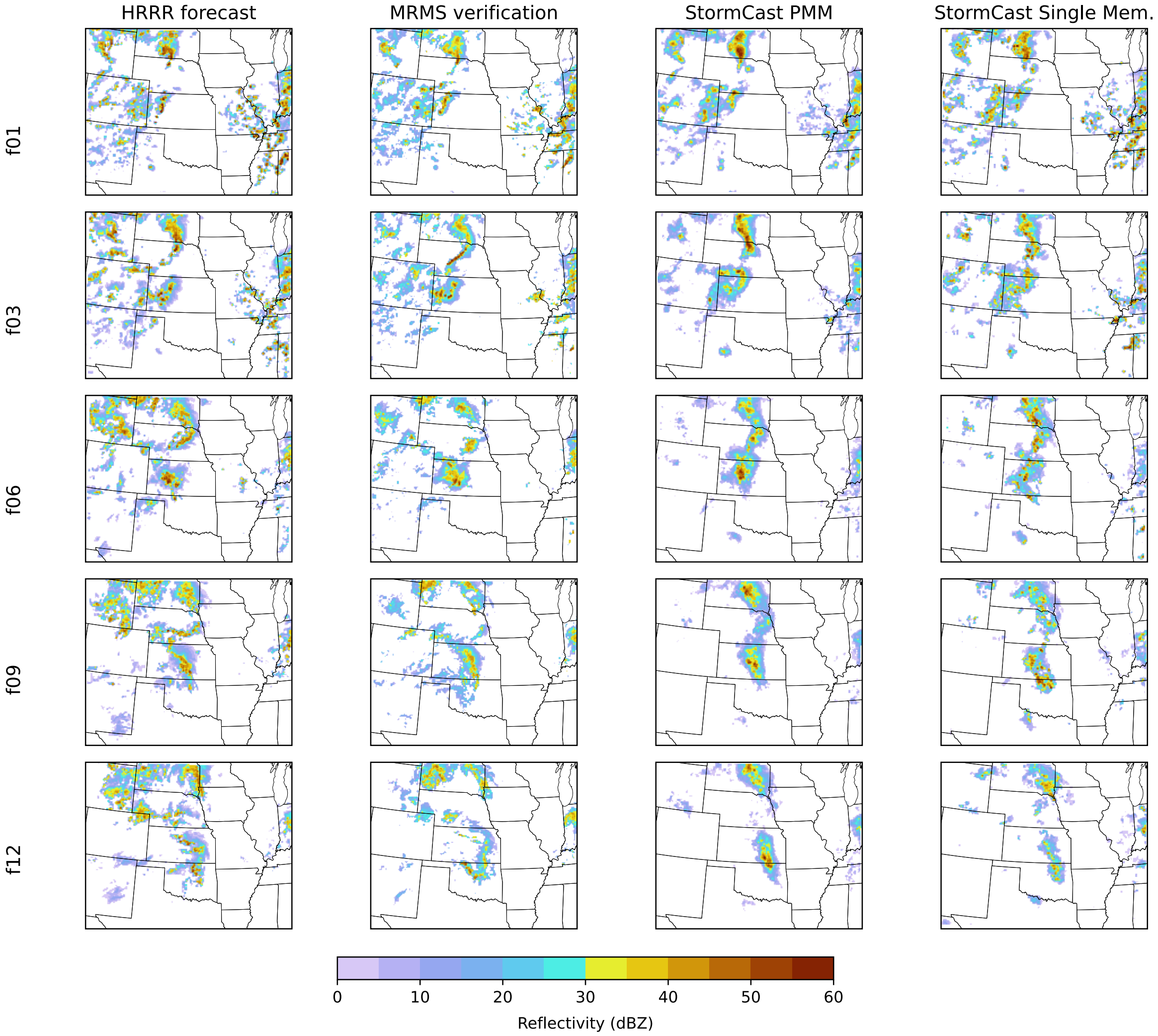


**Figure S17**: **Example forecast visualization from StormCast compared against MRMS verification and a corresponding HRRR forecast.** 2024-05-15 00:00:00 initialization

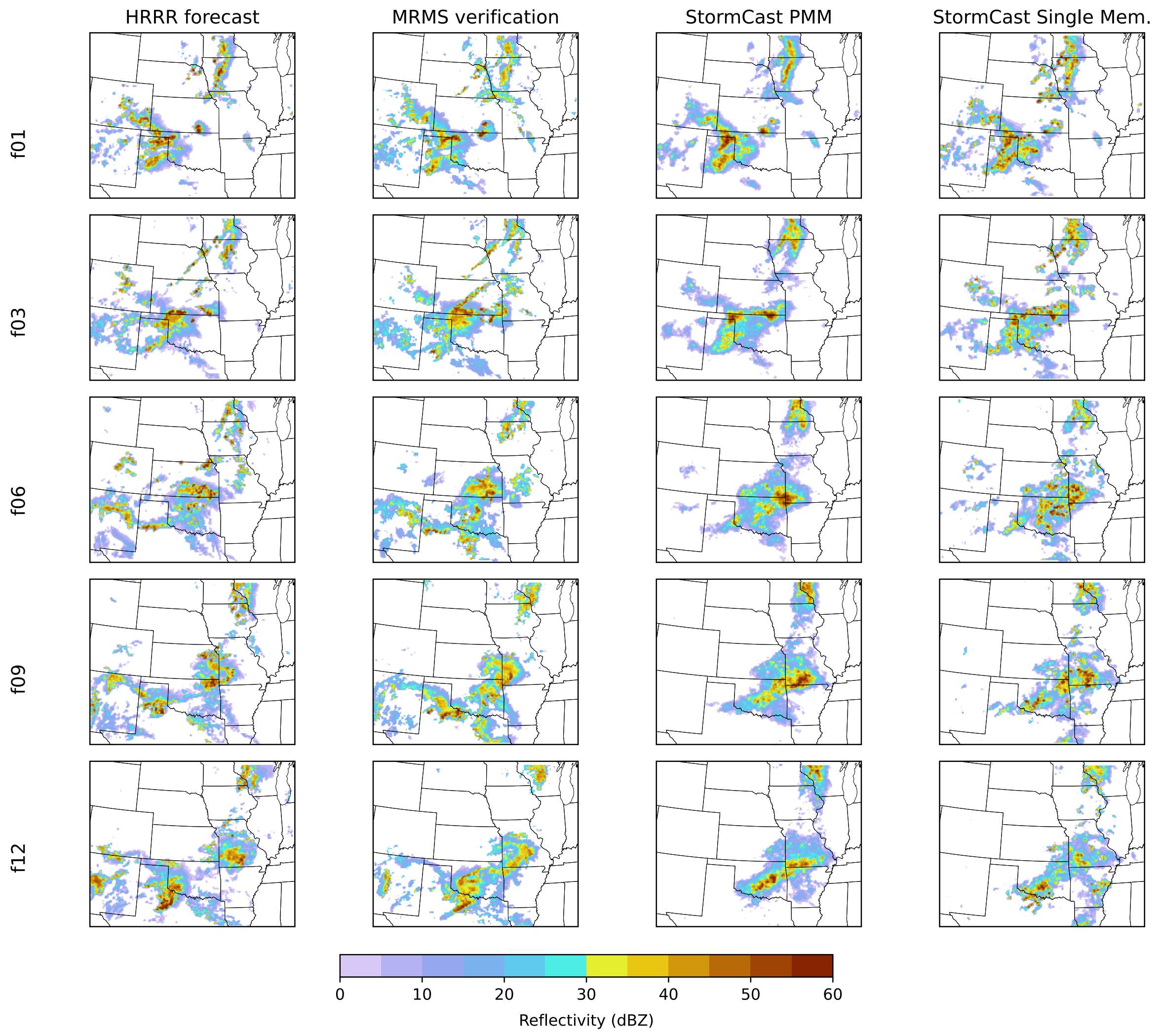


**Figure S18**: **Example forecast visualization from StormCast compared against MRMS verification and a corresponding HRRR forecast.** 2024-05-16 00:00:00 initialization

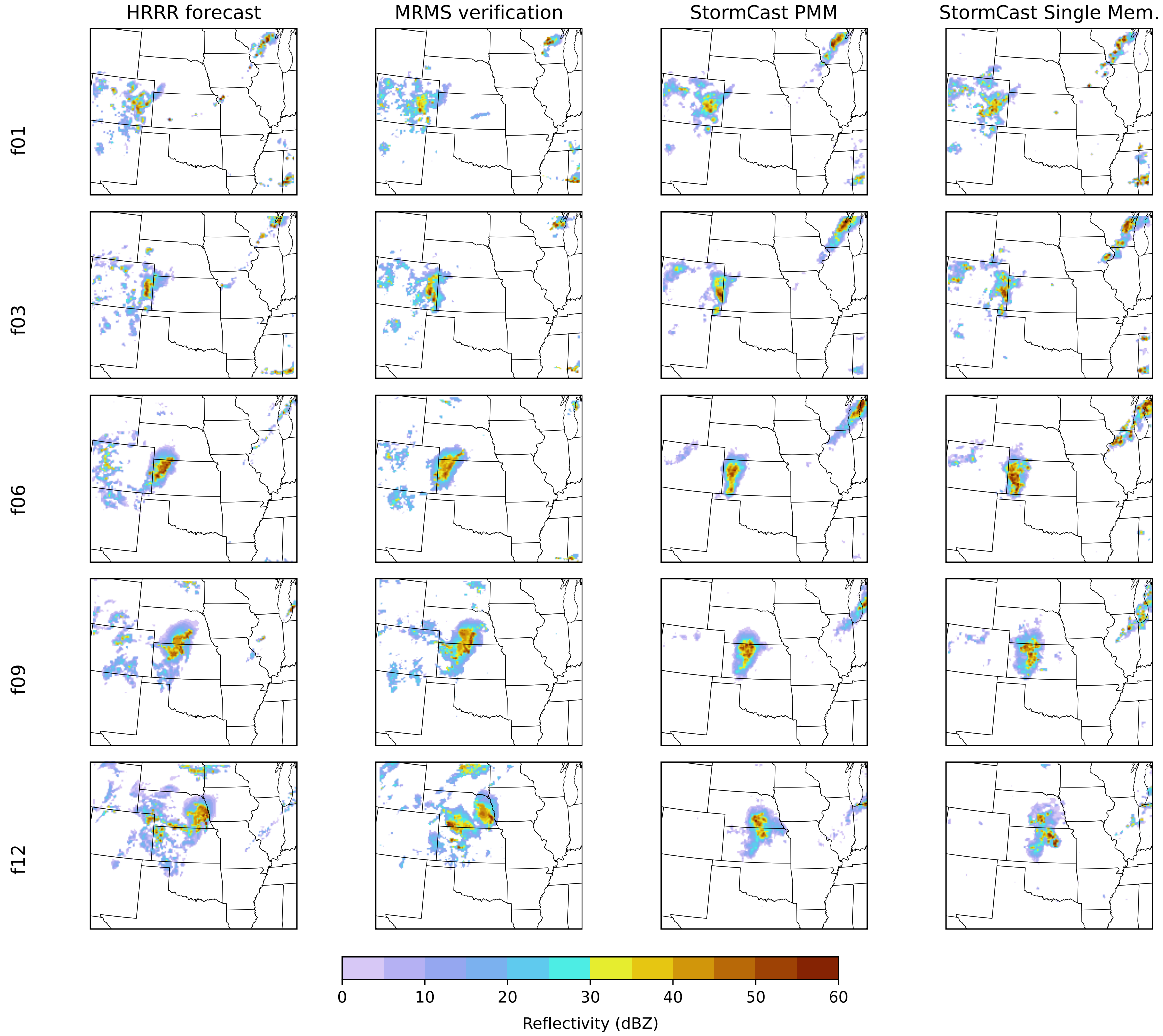


**Figure S19**: **Example forecast visualization from StormCast compared against MRMS verification and a corresponding HRRR forecast.** 2024-05-19 00:00:00 initialization

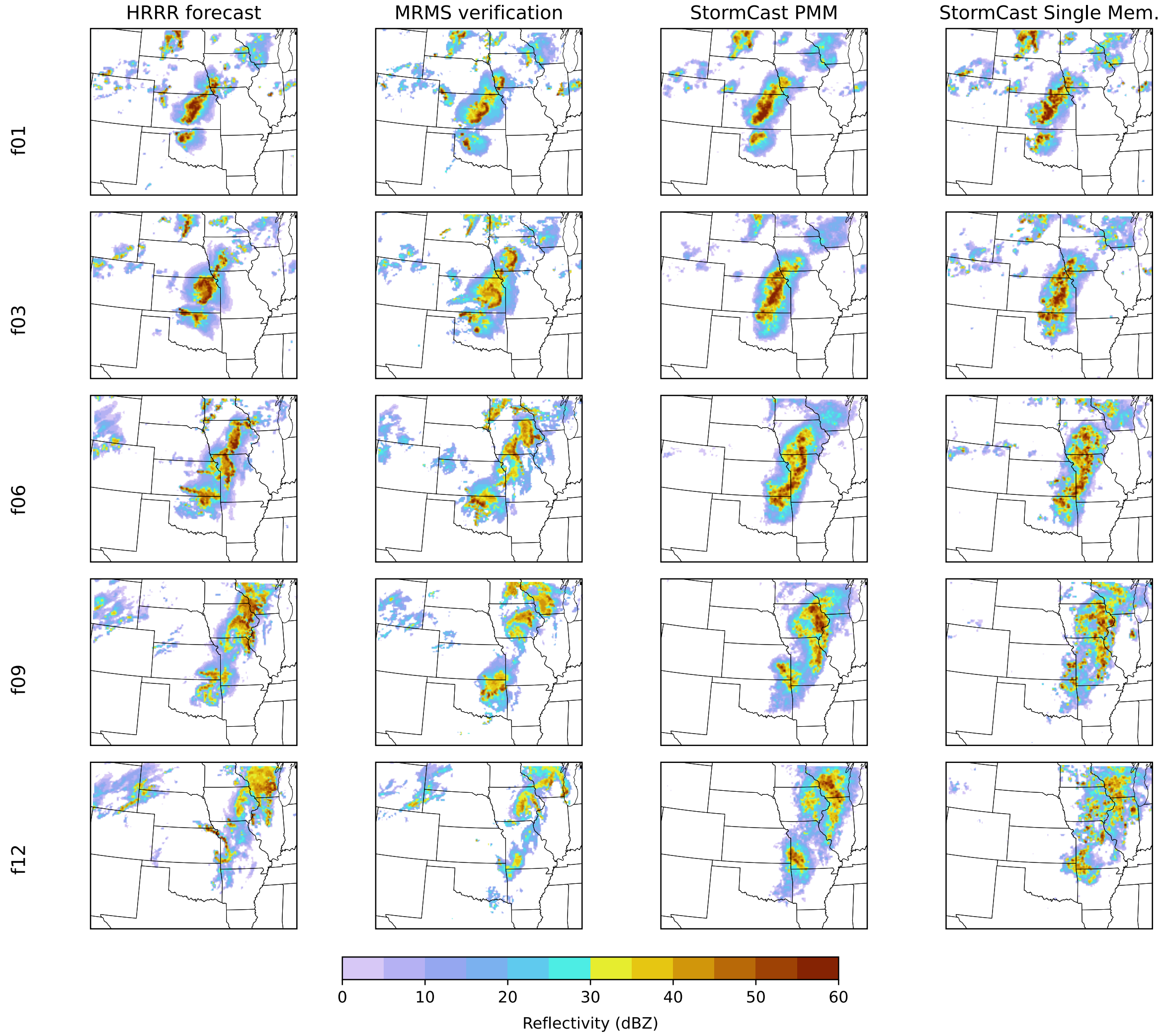


**Figure S20**: **Example forecast visualization from StormCast compared against MRMS verification and a corresponding HRRR forecast.** 2024-05-20 00:00:00 initialization

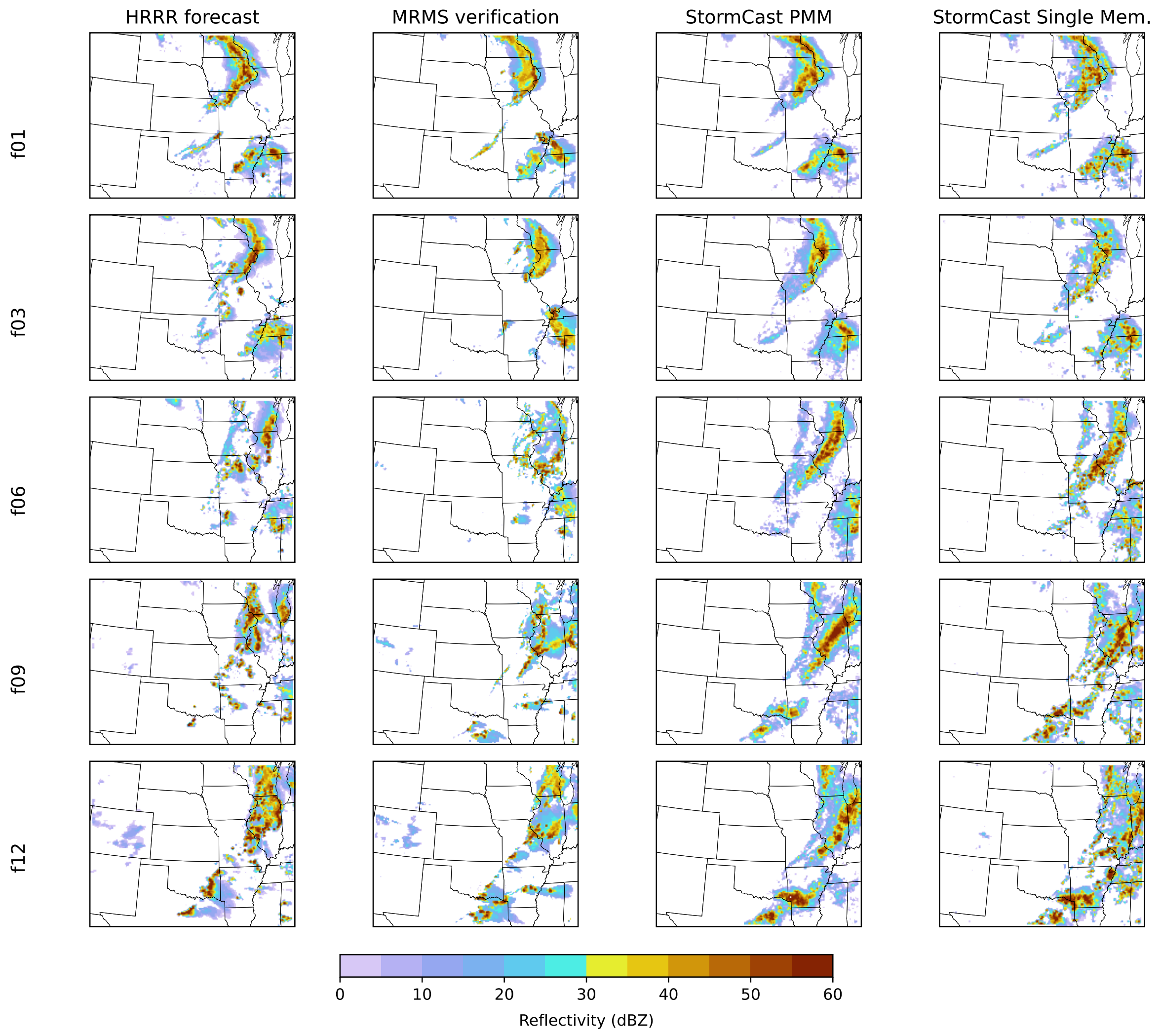


**Figure S21**: **Example forecast visualization from StormCast compared against MRMS verification and a corresponding HRRR forecast.** 2024-05-24 12:00:00 initialization

## REFERENCES


1. M. W. Moncrieff, “The multiscale organization of moist convection and the intersection of weather and climate” in *Climate Dynamics: Why Does Climate Vary?* (American Geophysical Union, 2010), vol. **189**, pp. 3–26.

2. P. Markowski, Y. Richardson, *Mesoscale Meteorology in Midlatitudes* (John Wiley & Sons, 2011).

3. NOAA National Centers for Environmental Information (NCEI), U.S. billion-dollar weather and climate disasters (2024); https://ncei.noaa.gov/access/billions/ [accessed 11 July 2024].

4. M. L Weisman, C. Davis, W. Wang, K. W Manning, J. B. Klemp, Experiences with 0–36-h explicit convective forecasts with the WRF-ARW model. *Weather Forecast.* **23**, 407–437 (2008).

5. W. C Skamarock, M. G. Duda, S. Ha, S.-H. Park, Limited-area atmospheric modeling using an unstructured mesh. *Mon. Weather Rev.* **146**, 3445–3460 (2018).

6. I.-H. Chen, J.-S. Hong, Y.-T. Tsai, C.-T. Fong, Improving afternoon thunderstorm prediction over taiwan through 3DVar-based radar and surface data assimilation. *Weather Forecast.* **35**, 2603–2620 (2020).

7. D. C. Dowell, C. R. Alexander, E. P James, S. S. Weygandt, S. G. Benjamin, G. S. Manikin, B. T. Blake, J. M. Brown, J. B. Olson, M. Hu, T. G. Smirnova, T. Ladwig, J. S. Kenyon, R. Ahmadov, D. D. Turner, J. D. Duda, T. I. Alcott, The High-Resolution RAPID REFRESH (HRRR): An hourly updating convection-allowing forecast model. Part I: Motivation and system description. *Weather Forecast.* **37**, 1371–1395 (2022).

8. E. P. James, C. R. Alexander, D. C. Dowell, S. S. Weygandt, S. G. Benjamin, G. S. Manikin, J. M. Brown, J. B. Olson, M. Hu, T. G. Smirnova, T. Ladwig, J. S. Kenyon, D. D. Turner, The High-Resolution Rapid Refresh (HRRR): An hourly updating convection-allowing forecast model. Part II: Forecast performance. *Weather Forecast.* **37**, 1397–1417 (2022).

9. P. Bauer, What if? Numerical weather prediction at the crossroads. arXiv:2407.03787 [physics.ao-ph] (2024).

10. T. C. Schulthess, P. Bauer, N. Wedi, O. Fuhrer, T. Hoefler, C. Schär, Reflecting on the goal and baseline for exascale computing: A roadmap based on weather and climate simulations. *Comput. Sci. Eng.* **21**, 30–41 (2018).

11.P. Bauer, A. Thorpe, G. Brunet, The quiet revolution of numerical weather prediction. *Nature* **525**, 47–55 (2015).

12. J. Shalf, The future of computing beyond Moore's law. *Philos. Trans. R. Soc. London Ser. A Math Phys Eng Sci.* **378**, 20190061 (2020).

13. J. Pathak, S. Subramanian, P. Harrington, S. Raja, A. Chattopadhyay, M. Mardani, T. Kurth, D. Hall, Z. Li, K. Azizzadenesheli, P. Hassanzadeh, K. Kashinath, A. Anandkumar, FourCastNet: A global data-driven high-resolution weather model using adaptive Fourier neural operators. arXiv:2202.11214 [physics.ao-ph] (2022).

14. B. Bonev, T. Kurth, C. Hundt, J. Pathak, M. Baust, K. Kashinath, A. Anandkumar, Spherical Fourier neural operators: Learning stable dynamics on the sphere. arXiv:2306.03838 [cs.LG] (2023).

15. R. Lam, A. Sanchez-Gonzalez, M. Willson, P. Wirnsberger, M. Fortunato, F. Alet, S. Ravuri, T. Ewalds, Z. Eaton-Rosen, W. Hu, A. Merose, S. Hoyer, G. Holland, O. Vinyals, J. Stott, A. Pritzel, S. Mohamed, P. Battaglia, Learning skillful medium-range global weather forecasting. *Science* **382**, 1416–1421 (2023).

16. K. Bi, L. Xie, H. Zhang, X. Chen, G. Xiaotao, Q. Tian, Accurate medium-range global weather forecasting with 3d neural networks. *Nature* **619**, 533–538 (2023).

17. S. Lang, M. Alexe, M. Chantry, J. Dramsch, F. Pinault, B. Raoult, M. C. A. Clare, C. Lessig, M. Maier-Gerber, L. Magnusson, Z. B. Bouallègue, A. P. Nemesio, P. D. Dueben, A. Brown, F. Pappenberger, F. Rabier, AIFS—ECMWF's data-driven forecasting system. arXiv:2406.01465 [physics.ao-ph] (2024).

18. I. Price, A. Sanchez-Gonzalez, F. Alet, T. R. Andersson, A. El-Kadi, D. Masters, T. Ewalds, J. Stott, S. Mohamed, P. Battaglia, R. Lam, M. Willson, Probabilistic weather forecasting with machine learning. *Nature* **637**, 84–90 (2025).

19. A. Mahesh, W. Collins, B. Bonev, N. Brenowitz, Y. Cohen, J. Elms, P. Harrington, K. Kashinath, T. Kurth, J. North, T. OBrien, M. Pritchard, D. Pruitt, M. Risser, S. Subramanian, J. Willard, Huge ensembles. Part I: Design of ensemble weather forecasts using spherical Fourier neural operators. arXiv:2408.03100 [physics.ao-ph] (2024).

20. A. Mahesh, W. Collins, B. Bonev, N. Brenowitz, Y. Cohen, P. Harrington, K. Kashinath, T. Kurth, J. North, T. OBrien, M. Pritchard, D. Pruitt, M. Risser, S. Subramanian, J. Willard, Huge ensembles. Part II: Properties of a huge ensemble of hindcasts generated with spherical Fourier neural operators. arXiv:2408.01581 [cs.LG] (2024).

21. T. Selz, M. Riemer, G. C. Craig, The transition from practical to intrinsic predictability of midlatitude weather. *J. Atmos. Sci.* **79**, 2013–2030 (2022).

22. K. V. Ooyama, Conceptual evolution of the theory and modeling of the tropical cyclone. *J. Meteorol. Soc. Jpn.* **60**, 369–380 (1982).

23. D. Raymond, Ž. Fuchs, S. Gjorgjievska, S. Sessions, Balanced dynamics and convection in the tropical troposphere. *J. Adv. Model. Earth Syst.* **7**, 1093–1116 (2015).

24. W. C. Skamarock, J. B. Klemp, J. Dudhia, D. O. Gill, D. M. Barker, M. G. Duda, X.-Y. Huang, W. Wang, J. G. Powers, A description of the advanced research WRF version 3 (NCAR Technical Note, National Center for Atmospheric Research, 2008), vol. **475**, pp. 10–5065).

25. M. Surcel, I. Zawadzki, M. K. Yau, On the filtering properties of ensemble averaging for storm-scale precipitation forecasts. *Mon. Weather Rev.* **142**, 1093–1105 (2014).

26. M. Surcel, I. Zawadzki, M. K. Yau, A study on the scale dependence of the predictability of precipitation patterns. *J. Atmos. Sci.* **72**, 216–235 (2015).

27. H. Hersbach, B. Bell, P. Berrisford, S. Hirahara, A. Horányi, J. Muñoz-Sabater, J. Nicolas, C. Peubey, R. Radu, D. Schepers, A. Simmons, C. Soci, S. Abdalla, X. Abellan, G. Balsamo, P. Bechtold, G. Biavati, J. Bidlot, M. Bonavita, G. De Chiara, P. Dahlgren, D. Dee, M. Diamantakis, R. Dragani, J. Flemming, R. Forbes, M. Fuentes, A. Geer, L. Haimberger, S. Healy, R. J. Hogan, E. Hólm, M. Janisková, S. Keeley, P. Laloyaux, P. Lopez, C. Lupu, G. Radnoti, P. de Rosnay, I. Rozum, F. Vamborg, S. Villaume, J.-N. Thépaut, The ERA5 global reanalysis. *Q. J. R. Meteorol. Soc.* **146**, 1999–2049 (2020).

28. J. Oskarsson, T. Landelius, F. Lindsten, Graph-based neural weather prediction for limited area modeling. arXiv:2309.17370 [cs.LG] (2023).

29. M. L. Flora, C. Potvin, WoFSCast: A machine learning model for predicting thunderstorms at watch-to-warning scales. Authorea Preprints (2024). https://doi.org/10.22541/essoar.172574503.30734251/v1.

30. M. Mardani, N. Brenowitz, Y. Cohen, J. Pathak, C.-Y. Chen, C.-C. Liu, A. Vahdat, M. A. Nabian, T. Ge, A. Subramaniam, K. Kashinath, J. Kautz, M. Pritchard, Residual corrective diffusion modeling for km-scale atmospheric downscaling. *Commun. Earth Environ.* **6**, 124 (2025).

31. J. Leinonen, D. Nerini, A. Berne, Stochastic super-resolution for downscaling time-evolving atmospheric fields with a generative adversarial network. *IEEE Trans. Geosci. Remote Sens.* **59**, 7211–7223 (2021).

32. S. Ravuri, K. Lenc, M. Willson, D. Kangin, R. Lam, P. Mirowski, M. Fitzsimons, M. Athanassiadou, S. Kashem, S. Madge, R. Prudden, A. Mandhane, A. Clark, A. Brock, K. Simonyan, R. Hadsell, N. Robinson, E. Clancy, A. Arribas, S. Mohamed, Skilful precipitation nowcasting using deep generative models of radar. *Nature* **597**, 672–677 (2021).

33. J. Leinonen, U. Hamann, D. Nerini, U. Germann, G. Franch, Latent diffusion models for generative precipitation nowcasting with accurate uncertainty quantification. arXiv:2304.12891 [physics.ao-ph] (2023).

34. C. K. Sønderby, L. Espeholt, J. Heek, M. Dehghani, A. Oliver, T. Salimans, S. Agrawal, J. Hickey, N. Kalchbrenner, MetNet: A neural weather model for precipitation forecasting. arXiv:2003.12140 [cs.LG] (2020).

35. M. Andrychowicz, L. Espeholt, D. Li, S. Merchant, A. Merose, F. Zyda, S. Agrawal, N. Kalchbrenner, Deep learning for day forecasts from sparse observations. arXiv:2306.06079 [physics.ao-ph] (2023).

36. L. Espeholt, S. Agrawal, C. Sønderby, M. Kumar, J. Heek, C. Bromberg, C. Gazen, R. Carver, M. Andrychowicz, J. Hickey, A. Bell, N. Kalchbrenner, Deep learning for twelve hour precipitation forecasts. *Nat. Commun.* **13**, 5145 (2022).

37. B. T. Smith, R. L. Thompson, J. S. Grams, C. Broyles, H. E. Brooks, Convective modes for significant severe thunderstorms in the contiguous United States. Part I: Storm classification and climatology. *Weather Forecast.* **27**, 1114–1135 (2012).

38. J. Sohl-Dickstein, E. Weiss, N. Maheswaranathan, S. Ganguli, “Deep unsupervised learning using nonequilibrium thermodynamics,” in *International Conference on Machine Learning* (PMLR, 2015), pp. 2256–2265.

39. J. Ho, A. Jain, P. Abbeel, “Denoising diffusion probabilistic models,” in *Proceedings of NeurIPS* (Association for Computing Machinery, 2020), vol. **33**, pp. 6840–6851.

40. A. Q. Nichol, P. Dhariwal, “Improved denoising diffusion probabilistic models,” in *International Conference on Machine Learning* (PMLR, 2021), pp. 8162–8171.

41. Y. Song, J. Sohl-Dickstein, D. P. Kingma, A. Kumar, S. Ermon, B. Poole, Score-based generative modeling through stochastic differential equations. arXiv:2011.13456 [cs.LG] (2020).

42. T. Karras, M. Aittala, T. Aila, S. Laine, Elucidating the design space of diffusion-based generative models. arXiv:2206.00364 [cs.CV] (2022).

43. N. M. Roberts, H. W. Lean, Scale-selective verification of rainfall accumulations from high-resolution forecasts of convective events. *Mon. Weather Rev.* **136**, 78–97 (2008).

44. I. Ebert-Uphoff, R. Lagerquist, K. Hilburn, Y. Lee, K. Haynes, J. Stock, C. Kumler, J. Q. Stewart, CIRA guide to custom loss functions for neural networks in environmental sciences—Version 1. arXiv:2106.09757 [cs.LG] (2021).

45. J. Zhang, K. Howard, C. Langston, B. Kaney, Y. Qi, L. Tang, H. Grams, Y. Wang, S. Cocks, S. Martinaitis, A. Arthur, K. Cooper, J. Brogden, D. Kitzmiller, Multi-Radar Multi-Sensor (MRMS) quantitative precipitation estimation: Initial operating capabilities. *Bull. Am. Meteorol. Soc.* **97**, 621–638 (2016).

46. R. N. Hoffman, E. Kalnay, Lagged average forecasting, an alternative to Monte Carlo forecasting. *Tellus A Dyn. Meteorol. Oceanogr.* **35**, 100–118 (2022).

47. R. E. Schlesinger, A numerical model of deep moist convection: Part I. Comparative experiments for variable ambient moisture and wind shear. *J. Atmos. Sci.* **30**, 835–856 (1973).

48. U. Schumann, C.-H. Moeng, Plume budgets in clear and cloudy convective boundary layers. *J. Atmos. Sci.* **48**, 1758–1770 (1991).

49. G. M. Barnes, Updraft evolution: A perspective from cloud base. *Mon. Weather Rev.* **123**, 2693–2715 (1995).

50. E. Kessler, Model of precipitation and vertical air currents. *Tellus* **26**, 519–542 (1974).

51. K. A. Emanuel, “Overview of atmospheric convection” in *The Physics and Parameterization of Moist Atmospheric Convection* (Springer, 1997), pp. 1–28.

52. S.-B. Park, T. Heus, P. Gentine, Role of convective mixing and evaporative cooling in shallow convection. *J. Geophys. Res. Atmos.* **122**, 5351–5363 (2017).

53. K. Sassen, Ice cloud content from radar reflectivity. *J. Clim. Appl. Meteorol.* **26**, 1050–1053 (1987).

54. C. A. Knight, L. J. Miller, First radar echoes from cumulus clouds. *Bull. Am. Meteorol. Soc.* **74**, 179–188 (1993).

55. A. M. Fridlind, A. S. Ackerman, A. Grandin, F. Dezitter, M. Weber, J. W. Strapp, A. V. Korolev, C. R. Williams, High ice water content at low radar reflectivity near deep convection—Part 1: Consistency of in situ and remote-sensing observations with stratiform rain column simulations. *Atmos. Chem. Phys.* **15**, 11713–11728 (2015).

56. NCAR MPAS Ensemble Forecasting Team, HWT 2024: MPAS ensemble forecasting at NCAR (2024); https://www2.mmm.ucar.edu/projects/ncar_ensemble/hwt2024_mpasens/about.php [accessed 30 June 2024].

57. C. Schwartz, R. A. Sobash, D. A. Ahijevych, L. M. Harris, K.-Y. Cheng, M. Morin, L. Zhou, "Evaluation of real-time, medium-range, convection-allowing ensemble forecasts produced for NOAA's 2023 Hazardous Weather Testbed Spring Forecasting Experiment," in *104th AMS Annual Meeting* (AMS, 2024).

58. I. Lopez-Gomez, Z. Y. Wan, L. Zepeda-Núñez, T. Schneider, J. Anderson, F. Sha, Dynamical-generative downscaling of climate model ensembles. arXiv:2410.01776 [physics.ao-ph] (2024).

59. H. Addison, E. Kendon, S. Ravuri, L. Aitchison, P. A. G. Watson, Machine learning emulation of precipitation from km-scale regional climate simulations using a diffusion model. arXiv:2407.14158 [physics.ao-ph] (2024).

60. Z. Y. Wan, I. Lopez-Gomez, R. Carver, T. Schneider, J. Anderson, F. Sha, L. Zepeda-Núñez, Statistical downscaling via high-dimensional distribution matching with generative models. arXiv:2412.08079v1 [cs.LG] (2024).

61. A. A. Saoulis, C. Lucas, N. S. Lord, N. Addor, J. S. Moraga, Diffusion models for climate data surpass alternative statistical downscaling techniques. Authorea Preprints (2025). https://doi.org/10.22541/essoar.173869444.40681416/v1.

62. R. M. Rasmussen, F. Chen, C. H. Liu, K. Ikeda, A. Prein, J. Kim, T. Schneider, A. Dai, D. Gochis, A. Dugger, Y. Zhang, A. Jaye, J. Dudhia, C. He, M. Harrold, L. Xue, S. Chen, A. Newman, E. Dougherty, R. Abolafia-Rosenzweig, N. D. Lybarger, R. Viger, D. Lesmes, K. Skalak, J. Brakebill, D. Cline, K. Dunne, K. Rasmussen, G. Miguez-Macho, CONUS404: The

NCAR–USGS 4-km long-term regional hydroclimate reanalysis over the CONUS. *Bull. Am. Meteorol. Soc.* **104**, E1382–E1408 (2023).

63. S. Lang, M. Alexe, M. C. A. Clare, C. Roberts, R. Adewoyin, Z. B. Bouallègue, M. Chantry, J. Dramsch, P. D. Dueben, S. Hahner, P. Maciel, A. Prieto-Nemesio, C. O'Brien, F. Pinault, J. Polster, B. Raoult, S. Tietsche, M. Leutbecher, AIFS-CRPS: Ensemble forecasting using a model trained with a loss function based on the continuous ranked probability score. arXiv:2412.15832 [physics.ao-ph] (2024).

64. N. Gustafsson, T. Janjić, C. Schraff, D. Leuenberger, M. Weissmann, H. Reich, P. Brousseau, T. Montmerle, E. Wattrelot, A. Bučánek, M. Mile, R. Hamdi, M. Lindskog, J. Barkmeijer, M. Dahlbom, B. Macpherson, S. Ballard, G. Inverarity, J. Carley, C. Alexander, D. Dowell, S. Liu, Y. Ikuta, T. Fujita, Survey of data assimilation methods for convective-scale numerical weather prediction at operational centres. *Q. J. R. Meteorol. Soc.* **144**, 1218–1256 (2018).

65. Y. Qu, J. Nathaniel, S. Li, P. Gentine, "Deep generative data assimilation in multimodal setting," in *Proceedings of the IEEE/CVF Conference on Computer Vision and Pattern Recognition* (IEEE/CVF, 2024), pp. 449–459.

66. L. Huang, L. Gianinazzi, Y. Yu, P. D. Dueben, T. Hoefler, DiffDA: A diffusion model for weather-scale data assimilation. arXiv:2401.05932 [cs.CE] (2024).

67. P. Manshausen, Y. Cohen, J. Pathak, M. Pritchard, P. Garg, M. Mardani, K. Kashinath, S. Byrne, N. Brenowitz, Generative data assimilation of sparse weather station observations at kilometer scales. arXiv:2406.16947 [cs.LG] (2024).

68. Y. Song, S. Ermon, "Generative modeling by estimating gradients of the data distribution," in *Advances in Neural Information Processing Systems (NeurIPS)* (Association for Computing Machinery, 2019).

69. J. Song, C. Meng, S. Ermon, "Denoising diffusion implicit models," in *Proceedings of the International Conference on Learning Representations (ICLR)* (ICLR, 2021).

70. L. Yang, Z. Zhang, Y. Song, S. Hong, X. Runsheng, Y. Zhao, W. Zhang, B. Cui, M.-H. Yang, Diffusion models: A comprehensive survey of methods and applications. *ACM Comput. Surv.* **56**, 1–39 (2024).

71. Y. Song, J. Sohl-Dickstein, D. P. Kingma, A. Kumar, S. Ermon, B. Poole, “Score-based generative modeling through stochastic differential equations,” in *Proceedings of the International Conference on Learning Representations (ICLR)* (ICLR, 2021).

72. National Oceanic and Atmospheric Administration (NOAA), NOAA Operational Model Archive and Distribution System (NOMADS) (2024); https://nomads.ncep.noaa.gov/ [accessed 29 July 2024].

73. E. E. Ebert, Ability of a poor man’s ensemble to predict the probability and distribution of precipitation. *Mon. Weather Rev.* **129**, 2461–2480 (2001).

74. A. J. Clark, Generation of ensemble mean precipitation forecasts from convection-allowing ensembles. *Weather Forecast.* **32**, 1569–1583 (2017).

75. C. S. Schwartz, G. S. Romine, K. R. Smith, M. L. Weisman, Characterizing and optimizing precipitation forecasts from a convection-permitting ensemble initialized by a mesoscale ensemble Kalman filter. *Weather Forecast.* **29**, 1295–1318 (2014).

76. A. J. Thorpe, M. J. Miller, M. W. Moncrieff, Two-dimensional convection in non-constant shear: A model of mid-latitude squall lines. *Q. J. R. Meteorol. Soc.* **108**, 739–762 (1982).

77. R. A. Houze Jr., A. K. Betts, Convection in gate. *Rev. Geophys.* **19**, 541–576 (1981).

78. T. Fujita, Precipitation and cold air production in mesoscale thunderstorm systems. *J. Atmos. Sci.* **16**, 454–466 (1959).

79. K. K. Droegemeier, R. B. Wilhelmson, Three-dimensional numerical modeling of convection produced by interacting thunderstorm outflows. Part I: Control simulation and low-level moisture variations. *J. Atmos. Sci.* **42**, 2381–2403 (1985).

80. A. M. Tompkins, Organization of tropical convection in low vertical wind shears: The role of cold pools. *J. Atmos. Sci.* **58**, 1650–1672 (2001).

81. Z. Feng, S. Hagos, A. K. Rowe, C. D. Burleyson, M. N. Martini, S. P. de Szoeke, Mechanisms of convective cloud organization by cold pools over tropical warm ocean during the AMIE/DYNAMO field campaign. *J. Adv. Model. Earth Syst.* **7**, 357–381 (2015).

82. L. D. Grant, B. Kirsch, J. Bukowski, N. M. Falk, C. A. Neumaier, M. Sakradzija, S. C. van den Heever, F. Ament, How variable are cold pools? *Geophys. Res. Lett.* **51**, e2023GL106784 (2024).

83. P. Garg, S. W. Nesbitt, T. J. Lang, G. Priftis, T. Chronis, J. D. Thayer, D. A. Hence, Identifying and characterizing tropical oceanic mesoscale cold pools using spaceborne scatterometer winds. *J. Geophys. Res. Atmos.* **125**, e2019JD031812 (2020).

84. W. Gao, X. Zhang, L. Yang, H. Liu, "An improved Sobel edge detection," in *2010 3rd International Conference on Computer Science and Information Technology* (IEEE, 2010), vol. **5**, pp. 67–71.

85. J. Baño-Medina, A. Sengupta, D. Watson-Parris, W. Hu, L. D. Monache, Towards calibrated ensembles of neural weather model forecasts. Authorea Preprints (2024). https://doi.org/10.22541/essoar.171536034.43833039/v1.
